# The glutamine synthetase of *Trypanosoma cruzi* is required for its resistance to ammonium accumulation and evasion of the parasitophorous vacuole during host-cell infection

**DOI:** 10.1371/journal.pntd.0006170

**Published:** 2018-01-10

**Authors:** Marcell Crispim, Flávia Silva Damasceno, Agustín Hernández, María Julia Barisón, Ismael Pretto Sauter, Raphael Souza Pavani, Alexandre Santos Moura, Elizabeth Mieko Furusho Pral, Mauro Cortez, Maria Carolina Elias, Ariel Mariano Silber

**Affiliations:** 1 Laboratory of Biochemistry of Tryps—LaBTryps, Department of Parasitology, Institute for Biomedical Sciences, University of Sao Paulo, São Paulo, Brazil; 2 Immunobiology of Leishmania-Macrophage Interaction Laboratory, Department of Parasitology, Institute for Biomedical Sciences, University of Sao Paulo, São Paulo, Brazil; 3 Special Laboratory of Cell Cycle, Center of Toxins, Immunology and Cell Signalling, Butantan Institute, São Paulo, SP, Brazil; Instituto de Ciências Biológicas, Universidade Federal de Minas Gerais, BRAZIL

## Abstract

*Trypanosoma cruzi*, the etiological agent of Chagas disease, consumes glucose and amino acids depending on the environmental availability of each nutrient during its complex life cycle. For example, amino acids are the major energy and carbon sources in the intracellular stages of the *T*. *cruzi* parasite, but their consumption produces an accumulation of NH_4_^+^ in the environment, which is toxic. These parasites do not have a functional urea cycle to secrete excess nitrogen as low-toxicity waste. Glutamine synthetase (GS) plays a central role in regulating the carbon/nitrogen balance in the metabolism of most living organisms. We show here that the gene *Tc*GS from *T*. *cruzi* encodes a functional glutamine synthetase; it can complement a defect in the *GLN1* gene from *Saccharomyces cerevisiae* and utilizes ATP, glutamate and ammonium to yield glutamine in vitro. Overall, its kinetic characteristics are similar to other eukaryotic enzymes, and it is dependent on divalent cations. Its cytosolic/mitochondrial localization was confirmed by immunofluorescence. Inhibition by Methionine sulfoximine revealed that GS activity is indispensable under excess ammonium conditions. Coincidently, its expression levels are maximal in the amastigote stage of the life cycle, when amino acids are preferably consumed, and NH_4_^+^ production is predictable. During host-cell invasion, *Tc*GS is required for the parasite to escape from the parasitophorous vacuole, a process *sine qua non* for the parasite to replicate and establish infection in host cells. These results are the first to establish a link between the activity of a metabolic enzyme and the ability of a parasite to reach its intracellular niche to replicate and establish host-cell infection.

## Author summary

*Trypanosoma cruzi*, the agent that causes Chagas disease, has a complex life cycle, alternating between insects and mammals and thus facing environments with different metabolite compositions. *T*. *cruzi* can consume glucose and/or amino acids, depending on their availability. However, amino acid consumption produces excess ammonium, which must be eliminated in a non-toxic manner. Here, we show that the enzyme glutamine synthetase contributes to the management of excess ammonium and uses it to form the amino acid glutamine. During its life cycle, the parasite invades mammalian host cells and transiently becomes enclosed in a tight vacuole, where it differentiates into the amastigote, an amino acid consumer stage. Amastigotes must escape from the vacuole into the host-cell cytoplasm to initiate intracellular replication. In this work, we show that the inhibition of *T*. *cruzi* glutamine synthetase aborts parasite evasion from the vacuole. We propose that this enzyme contributes to the control of ammonium produced by parasite metabolism, as ammonium increases the internal pH of the parasitophorous vacuole, making the enzymes for the *T*. *cruzi* evasion process non-functional. This knowledge could be useful for designing new anti-*T*. *cruzi* drugs.

## Introduction

Parasites display metabolic peculiarities that help them adapt to different environments during their life cycles and take advantage of the host’s resources. *Trypanosoma cruzi*, the causative agent of Chagas disease, is a digenetic protozoan that transitions among different environments in its vertebrate and invertebrate hosts during its life cycle, alternating between non-replicative and replicative stages [1]. Briefly, epimastigotes (E), which are the replicative forms in the insect vector, colonize the digestive tube and differentiate into non-dividing infective metacyclic trypomastigotes (MT) in its terminal portion [2]. MT must invade the mammalian host cells through an energy-dependent mechanism to be able to differentiate into replicative stages and establish infection [3,4]. The trypomastigote invasion of host cells is an event that involves the recruitment of lysosomes to form a parasitophorous vacuole [5]. Once inside, it is assumed that low pH triggers the differentiation of MT into replicative intracellular amastigotes (A) [6], which activates hydrolytic activities, enabling the release of A into the cytoplasm to initiate their replication [7–10]. After a variable number of cell divisions, A differentiate into a transient replicative form called intracellular epimastigotes (IE), which ultimately differentiate into cell-derived trypomastigotes (CDT) [11]. CDT lyse the infected cells, and once they burst, the CDT have two fates: i. to infect neighbor cells; and ii. to reach the bloodstream, from which they can reach and infect remote tissues, or if a triatomine makes a bloodmeal while the CDT are circulating, they can infect a new insect, which will transmit the parasite into new mammalian hosts [6].

*T*. *cruzi* faces different physicochemical and nutritional conditions during its complex journey among different hosts. For example, it is well known that during midgut colonization, E preferably consume glucose during exponential growth and switches to the consumption of amino acids in the stationary phase [12,13]. An orchestrated metabolic switch happens: while the uptake of amino acids and several of their intermediate metabolites increases, the level of most glycolysis metabolites diminishes [14]. In addition, the A and IE stages obtain energy predominantly from amino acids during intracellular life [15], whereas glucose seems to represent a significant energy source in only the form of CDT [16]. In summary, the *T*. *cruzi* life cycle involves plenty of situations in which glucose is scarce, and there is solid evidence showing amino acid consumption as an alternative energy source [17].

A main waste product of amino acid catabolism in ammoniotelic organisms is reduced metabolites containing -NH_2_ groups and NH_3_, which is spontaneously converted into NH_4_^+^ in aqueous media. It is well known that *T*. *cruzi* does not have a functional urea cycle: alanine and NH_3_ are known nitrogen-containing excreta products [18–21]. The management of excesses of these compounds requires an enzymatic system that is able to recover NH_4_^+^ from H_2_O (such as reversible, non-oxidative glutamate dehydrogenases) and a robust transaminase network [18]. In other words, a specific metabolic configuration is required to address NH_4_^+^ accumulation in organisms that are avid amino acid consumers without a urea cycle. In this regard, a robust transamination network was described (at least for E) to transfer -NH_2_ from amino acids to oxoacids (primarily but not exclusively α-ketoglutarate and oxaloacetate, rendering glutamate and aspartate, respectively, which are donors of -NH_2_ in a transamination reaction with pyruvate as the acceptor, forming Ala) [22]. Eventually, if an increase in the ratio of glutamate/α-ketoglutarate occurs, two isoforms of glutamate dehydrogenase can also reversibly transfer the -NH_2_ group of glutamate to H_2_O, forming NH_4_^+^ [23–25] (also reviewed in [17,18]). However, it should be noted that this step goes back to the initial problem of NH_4_^+^ accumulation, and in this situation, this reaction would stop or even go backward. Thus, an alternative step allowing the capture of NH_4_^+^ may be essential in these organisms.

Glutamine synthetase (GS) [L-glutamate-ammonia ligase; EC 6.3.1.2] catalyzes the ATP-dependent formation of glutamine from glutamate and ammonia. GS has a major role in all organisms studied thus far. In particular, GS is the major NH_4_^+^-assimilation pathway in most organisms and is the enzyme that controls carbon/nitrogen balance in plants and animals alike [26–28], simultaneously playing an important role in the maintenance of low concentrations of toxic ammonia in the mitochondria of plants [29,30] and uricotelic vertebrates [31]. In mammals, its role in NH_4_^+^ detoxification is also well established for several tissues, including brain and muscle [32]. It has received extensive attention for many years as a central metabolic point in both eukaryotes and prokaryotes. Thus, its structure, allosteric interactions and the effect of inhibitors on its activity are well characterized in species throughout nearly the entire range of living organisms, such as bacteria [33,34], cyanobacteria [35], fungi and yeast [30,36], insects [37] and mammals [38,39]. It is known that GSs are present in trypanosomatids; an active recombinant GS was obtained after cloning and expressing of the corresponding gene from *Leishmania donovani* [40], and GS activity was observed in *T*. *cruzi* E cell-free extracts [41,42]. However, the molecular and cellular aspects of the Glutamate—Glutamine pathway or its components have not been investigated in these pathogenic organisms. In this work, we present the first molecular and enzymological characterization of a GS from a trypanosoma. The data presented here show that GS in *T*. *cruzi* is fully functional in both of its localizations, similar to GS in plants and uricotelic vertebrates. We also show that this enzyme is involved in ammonia detoxification. Furthermore, we show for the first time that this enzyme affects the intracellular life stages of *T*. *cruzi* and is critical for its escape from parasitophorous vacuoles, which is required for the parasite to initiate intracellular replication.

## Results

### The gene *Tc*GS encodes an octameric glutamine synthetase

**Fig 1:**
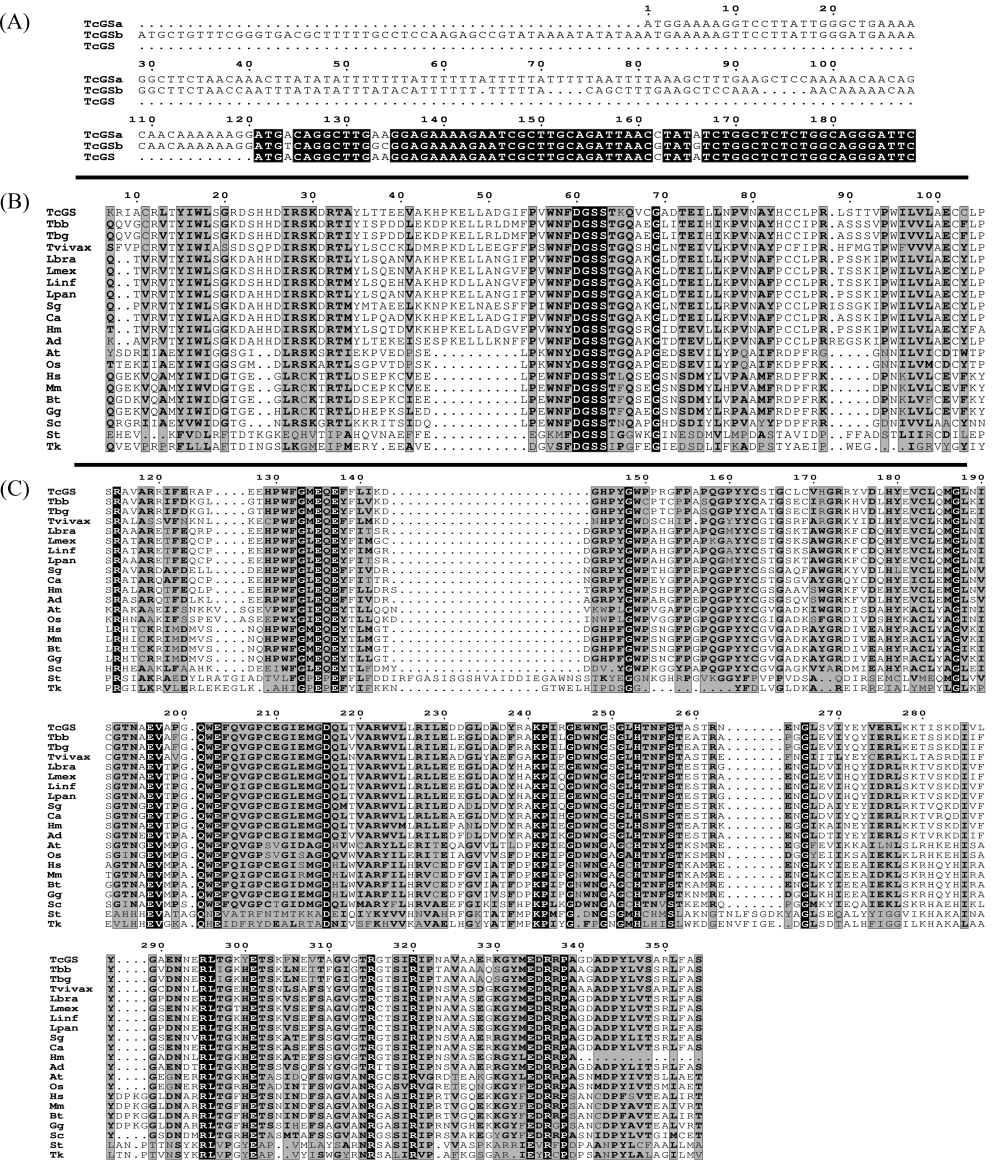
(A) Transmembrane domain predicted by Phobius [43] in *Tc*GSa: TcCLB.508175.370; *Tc*GSb: TcCLB.503405.10 and *Tc*GS, the sequence used in this work; (B) β-grasp domain predicted by Pfam [44]; (C) Catalytic domain predicted by Pfam. The amino acid sequence of *Tc*GS was aligned to orthologs from Tbb: *Trypanosoma brucei* brucei; Tbg: *Trypanosoma brucei* gambiense; Tvivax: *Trypanosoma vivax;* Lbra: *Leishmania brasiliensis*; Lmex: *Leishmania mexicana*; Linf: *Leishmania infantum*; Lpan: *Leishmania panamensis*; Sg: *Strigomonas galati*; Ca: *Crithidia acanthocephala*; Hm: *Herpetomonas muscarum*; Ad: *Angomonas deanei*; At: *Arabidopsis thaliana*; Oa: *Oryza sativa*; Hs: *Homo sapiens*; Mm: *Mus musculus*; Bt: *Bos taurus*; Gg: *Gallus*; St: *Salmonella typhimurium*; Sc: *Saccharomyces cerevisiae*; St: *Salmonella typhimurium*; and Tk: *Thermococcus kodakarensis*.

As mentioned, the existence of GS activity in *T*. *cruzi* was previously shown [41,42]. We further characterized this *T*. *cruzi* enzyme by initially identifying two sequences in both haplotypes (Esmeraldo-like and Non-Esmeraldo like) of the *T*. *cruzi* CL Brener strain genome (TcCLB.503405.10 –Esmeraldo-like, seq. a- and TcCLB.508175.370 –Non-Esmeraldo-like, seq. b-) encoding putative type II glutamine synthetases (GSII) (Fig 1). Both sequences are located on chromosome 27, spanning from coordinate 561646 to either 562911 (seq. a) or 562953 (seq. b). They both display typical signatures of GSs. Allelic variants were also found in gene databases for the other strains. Sequences a and b also had an unusual 5’ terminus, consisting of A- and T-enriched stretches that encode a predicted transmembrane domain (Fig 1A). This region is absent in the sequences of *T*. *cruzi* DM28 and Marinkellei strains and in any other GSs that has been studied or annotated so far. We clarified this issue by sequencing the genome fragments corresponding to seq. a and seq. b from the low-infectivity strain CL14. These sequences were identical to those reported for the closely related strain CL Brener, and they revealed that the predicted translation start is out-of-frame for a functional GS, whereas an ATG codon lying 123 base pairs downstream conformed to a canonical GS open reading frame (ORF). For these reasons, we established that the 5’ regions predicted in both seq. a and b from the CL Brener strain were not actually parts of the gene (Fig 1B). We named this gene *Tc*GS and selected sequence TcCLB.503405.10 (seq. a, Esmeraldo-like haplotype from CL Brenner) starting at base pair 123 as our reference allele. A blast search for other GS genes in the *T*. *cruzi* genome yielded no other sequences. Phylogenetic analysis of the sequence revealed that it was closely related to sequences found in other trypanosomatids and the *Leishmania* genus. It was also shown to be related to other eukaryotic GS genes (Fig 1C).

**Fig 2:**
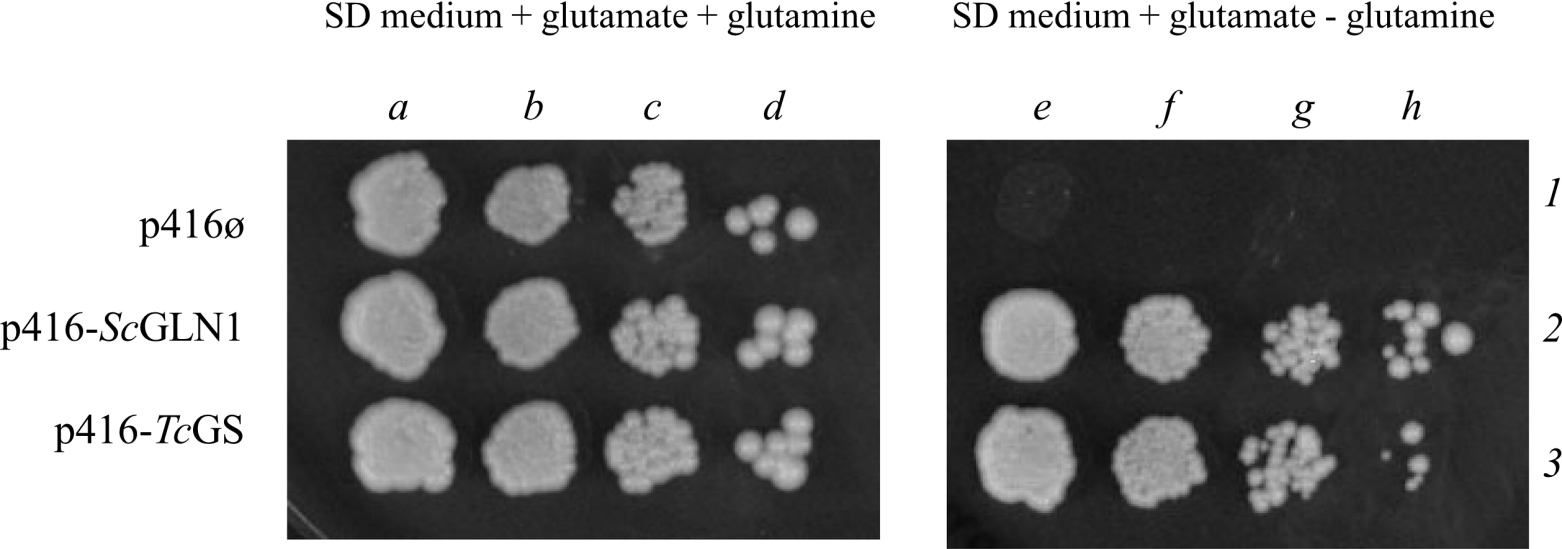
Yeast functional complementation assay. *Saccharomyces cerevisiae* SAH35 yeast with an endogenous glutamine synthetase gene controlled by the *GAL1* gene promoter was transformed with an empty plasmid, a copy of *S*. *cerevisiae* GS gene or the *T*. *cruzi* gene (p416, p416-*Sc*GLN1 and p416-*Tc*GS, respectively) and plated at different dilutions (a and e: 10^4^ yeasts; b and f: ≈10^3^ yeasts; c and g: ≈100 yeasts d and h: ≈10 yeasts). The endogenous *ScGLN1* gene is not transcribed in a defined medium with glutamate as a *ScGLN1* non-repressible nitrogen source and with glucose as a carbon source. Furthermore, when glutamine is supplied, compensation of the glutamine biosynthesis pathway occurs; GS is not required in this case, and it is not essential for yeast clones (line 1—a to d). However, the empty vector transformed yeast do not grow in a medium without glutamine (line 1—e to h). The yeast recover the capacity to proliferate when they are transformed with the same gene (*ScGLN1*) or with GS from *T*. *cruzi*.

We performed a functional complementation assay in *Saccharomyces cerevisiae* to evaluate the ability of the *Tc*GS gene to express a functional enzyme and support cell growth by providing glutamine. We first amplified the *Tc*GS ORF by high fidelity PCR and then sequenced and cloned expression vector p416GPD, a centromeric plasmid, into yeast as described in the Materials and Methods. As GS is essential for yeast in the absence of glutamine under most usual growth conditions, we chose to transfect the construction into strain SAH35, a conditional mutant for *ScGLN1* that does not express endogenous GS when grown on glucose as a carbon source. In other words, in the absence of glucose, both, the endogenous version of GS and TcGS are expressed, but in the presence of glucose, only TcGS is expressed. Therefore, for our system, in the absence of glutamine and the presence of glucose, growth rely exclusively on the ability of TcGS to encode a functional GS. The expression of gene *Tc*GS was able to restore SAH35 growth in the presence of glucose in a similar way to the reintroduction of *ScGLN1* (Fig 2), confirming that this gene encodes a functional GS.

**Fig 3:**
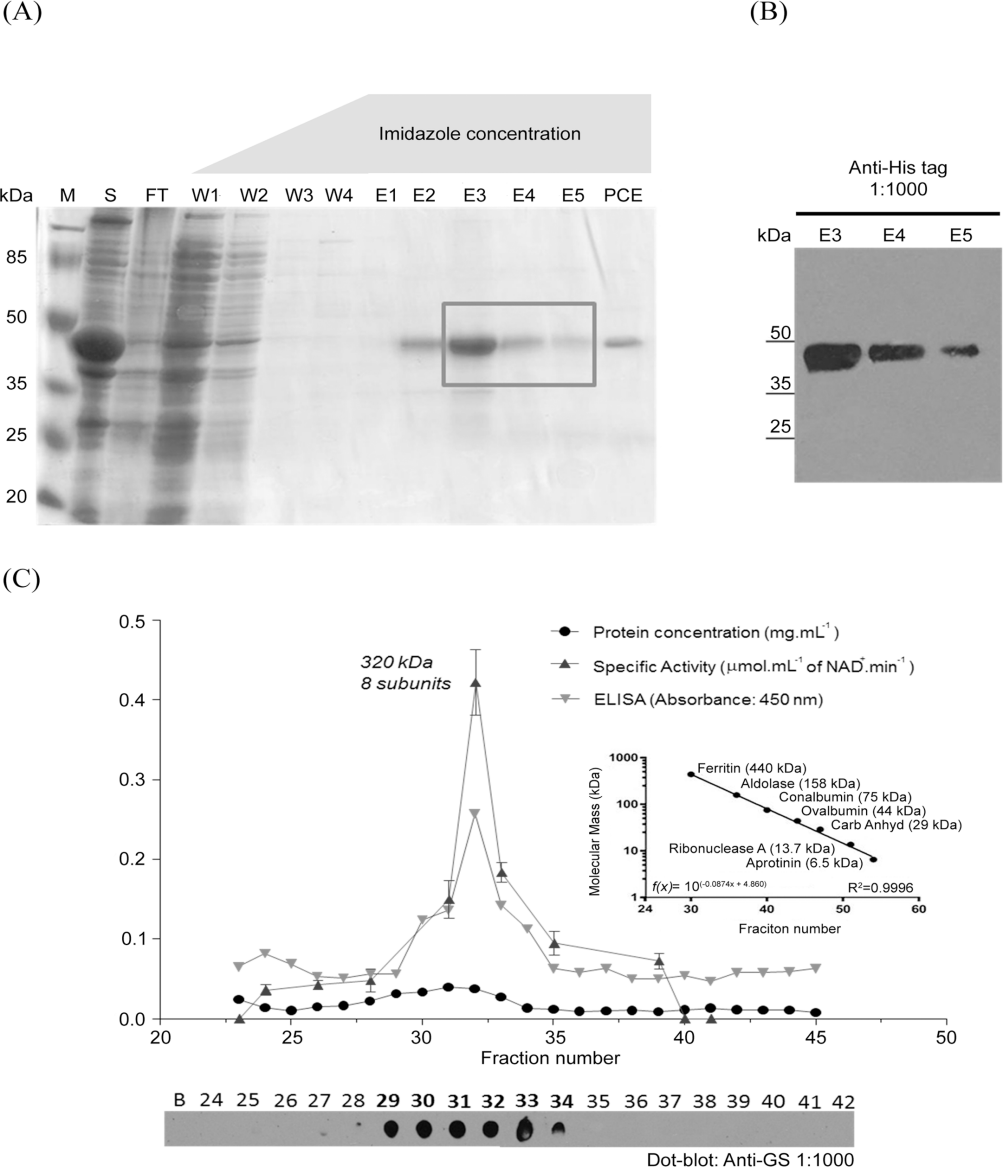
Heterologous expression and purification of recombinant *Tc*GS. **(A)** The recombinant protein was analyzed by SDS-PAGE using 10% (v/v) polyacrylamide gels under reducing conditions and visualized by Coomassie Blue staining. *M*: molecular mass maker; *S*: Supernatant of lysed bacterial culture overexpressing *Tc*GS; *FT*: Supernatant after flowing through the column; *W1 to W4*: Samples of column washes with buffers with crescent concentrations of imidazole (5 to 100 mM); *E1 to E5*: Elution fractions performed with a buffer containing 500 mM imidazole; *PCE*: Fraction after passing through Amicon Ultra Centrifugal filters with a 30,000-Da cut-off. **(B)** Western blot analysis was performed using an anti-His_6_ antibody raised against the recombinant enzyme elutions E3 to E5, as indicated by the box. (C) Size-exclusion chromatography (SEC) of the elutions. Four methodologies were applied to estimate the presence of *Tc*GS. They are listed as follows in ascending order of specificity: a Bradford assay quantifying the total content of protein in the samples; a dot-blot identifying a *Tc*GS signal in 6 SEC fractions (*bottom*); an ELISA assay quantifying the total amount of *Tc*GS in the fractions; and a GS activity assay showing the functionality of *Tc*GS in different oligomeric conformations. Error bars represent standard deviation (n = 3). *Inset*: Calibration of the SEC assay utilized to estimate the number of subunits of the sample.

Once the functionality of the *Tc*GS product was confirmed, we decided to better characterize it by expressing the active recombinant protein. We cloned *Tc*GS into the bacterial expression vector pET-28a(+), which also provided a His_6_ tag for purification. The purified protein showed an apparent molecular mass of 45 kDa, which is close to the predicted mass (42 kDa) (Fig 3A) when evaluated by SDS-PAGE. However, when the soluble native protein from bacterial extracts was analyzed by analytical size-exclusion chromatography, the estimated molecular mass was 320 kDa (Fig 3B), pointing to an octameric conformation of the native protein.

### Biochemical characterization of *Tc*GS

**Fig 4:**
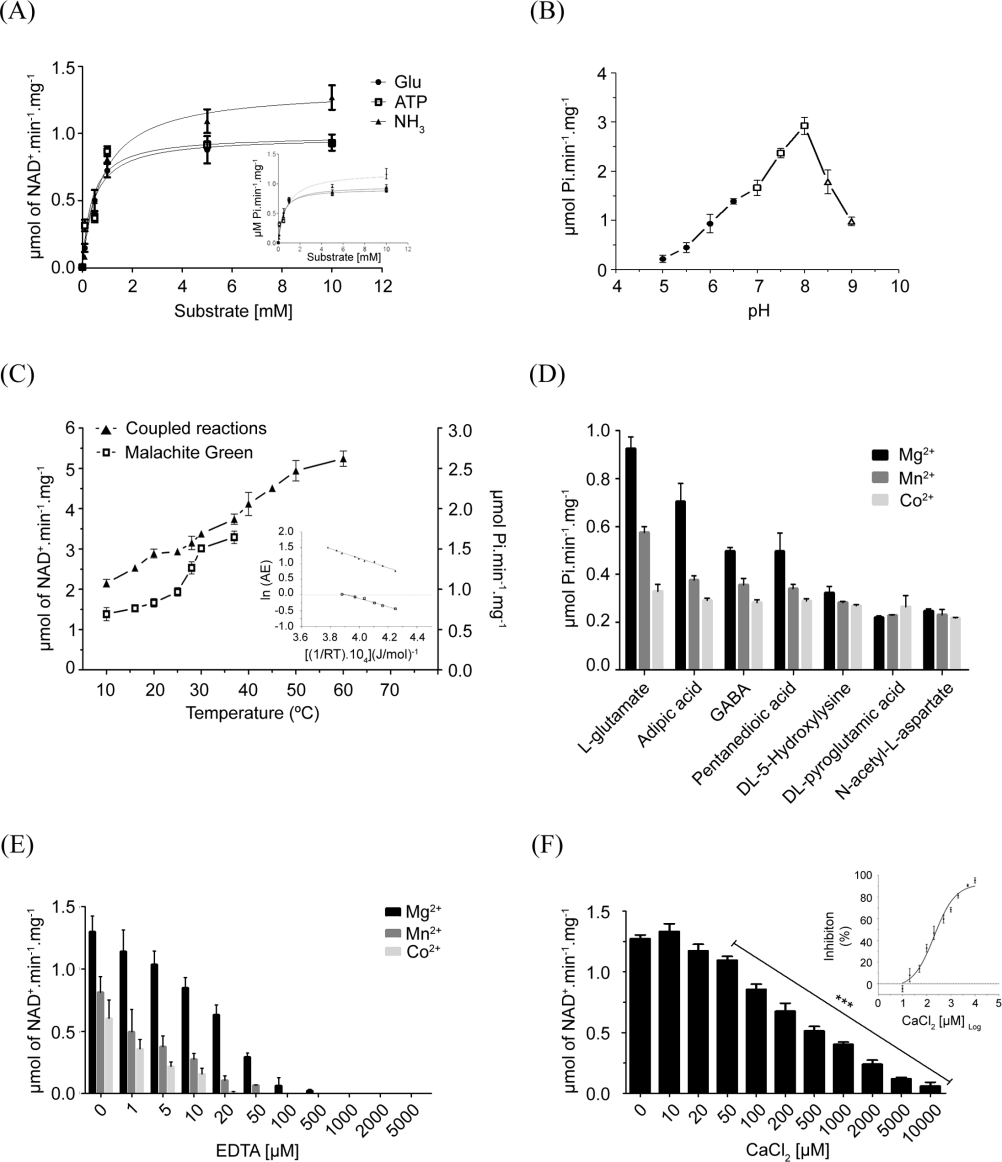
Effect of substrate, pH and temperature variations on *Tc*GS activity. (A) The coupled reactions and malachite green methods were applied to access the kinetic parameters V_max_ and K_M_ related to the three substrates of *Tc*GS (Glutamate, ATP and NH_3_); data were adjusted to a Michaelis-Menten equation. (B) The pH of the media in the reaction catalyzed by *Tc*GS was modified using different buffer systems. Enzymatic activity was determined in the presence of 1 mM glutamate, 1 mM ATP, 2 mM NH_4_Cl and 100 mM of reaction buffer as follows: MES NaOH (pH 5.0 to 6.5) (filled circles), imidazole HCl (pH 7.0 to 8.0) (open squares), and Tris-HCl (pH 8.5 to 9.0) (open triangles). The reaction was initiated by the addition of the enzyme, and the initial velocities were calculated as linear rates for *Tc*GS-His_6_. (C) Enzymatic activity was determined by progressively increasing the reaction temperature (from 10 to 60°C). Inset: The activation energy values were estimated by an Arrhenius plot of the specific activity of *Tc*GS. y-axis: log of GS activity according to tested temperature values; x-axis: (molar gas constant x temperature values)^-1^ x (temperature in Kelvin). (D) *Tc*GS activity was measured in the presence of the three divalent ions (Mg^2+^, Mn^2+^ or Co^2+^), and the effect on activity caused by the replacement of the natural substrate of the enzyme (L-glutamate) by other amino acids was evaluated. Saturating concentrations were used for two GS substrates (NH_3_ and ATP) and a 1 mM concentration of each glutamate analog. (E) Effect of increasing the EDTA concentration on *Tc*GS activity. (F) Effect of increasing Ca^2+^ concentration on *Tc*GS activity under standard conditions. *Inset*: Dose-response curve of Ca^2+^; IC_50_ = 205.7 ± 2.8 μM. The values of the enzymatic parameters are available in Table 1. Statistical analysis were made using one-way ANOVA / Dunnet’a Multiple Comparison Test. *p<0.05, **p<0.01, ***p<0.001. Error bars represent standard deviation (n = 3).

*Tc*GS expressed from *E*. *coli* was used for kinetic and enzymological characterizations. The reaction catalyzed by the recombinant enzyme was dependent on L-glutamate, NH_4_^+^ and ATP concentrations (Fig 4A) and showed an optimal pH at 8.0 (Fig 4B). The K_M_ of each of the substrates was in the submillimolar range (Table 1). Kinetic data were used to obtain the catalytic constants of the recombinant enzyme, including the activation energy of the reaction (Fig 4C).

**Table 1:** Enzymatic characterization overview.

	Substrate	V_max_^1^ (μmol NAD^+^.min^-1^.mg^-1^ or nmol P_i_.min^-1^.mg^-1^)	K_M_^2^ (mM)	K_cat_^3^ (s^-1^)	K_cat_/K_M_^4^ (M^-1^. s^-1^)	E_a_^5^ (KJ. mol^-1^)	Optimum pH
**Recombinant enzyme** **(Coupled reactions assay)**	**Glu**	0.97 ± 0.03	0.44 ± 0.06	392 ± 9.5	8.9 x 10^5^	1.304	n.m.
	**ATP**	0.98 ± 0.05	0.38 ± 0.10	396 ± 18.2	10.5 x 10^5^		
	**NH**_**4**_^**+**^	1.33 ± 0.04	0.79 ± 0.10	535 ± 15.6	6.8 x 10^5^		
**Recombinant enzyme** **(Malachite green assay)**	**Glu**	0.91 ± 0.02	0.47 ± 0.05	357 ± 12.1	8.6 x 10^5^	1.515	8.0
	**ATP**	0.91 ± 0.05	0.39 ± 0.09	359 ± 22.9	9.3 x 10^5^		
	**NH**_**4**_^**+**^	1.20 ± 0.09	0.78 ± 0.10	480 ± 17.5	6.2 x 10^5^		
**Epimastigote extract** **(Coupled reactions assay)**	**Glu**	1.06 ± 0.03	0.32 ± 0.04	n.m.	n.m.	n.m.	n.m.
	**ATP**	2.16 ± 0.05	0.203 ± 0.034	n.m.			
	**NH**_**4**_^**+**^	1.54 ± 0.05	0.698 ± 0.012	n.m.			

The parameters were established using the recombinant enzyme (*Tc*GSr) and epimastigote extracts. Two protocols were applied with *Tc*GSr: malachite green assay and coupled reactions assay. Glu: glutamate. (Values corresponding to *Tc*GS were obtained from the data in Fig 4, and values corresponding to GS from E cell-free extracts were obtained from data in S1 Fig).

The ATPase activity of *Tc*GS was tested for all amino acids and was found to be specific for glutamate; aspartate, asparagine or histidine, however, the last three supported less than 10% of the activity observed with glutamate. All the other amino acids did not promote ATP hydrolysis. In contrast, glutamate analogs could successfully drive ATPase activity, although to various extents. Thus, while we observed nearly 75% activity with adipic acid, γ-aminobutyric acid (GABA) or pentanedioic acid was able to produce only 50%. Other analogs were less effective (Fig 4D). The activity was dependent on the presence of divalent cations. Mg^2+^ was the most effective cation to support GS activity, but Mn^2+^ and Co^2+^ were able to support activity levels above 50% compared with magnesium in standard conditions of substrates, temperature and pH (Fig 4E). Almost no activity was found in the presence of Zn^2+^ ions, such as in the presence of the divalent metal chelator EDTA. Ca^2+^ was a special case. No activity was found in the presence of Ca^2+^ alone. In addition, Ca^2+^ showed an inhibitory effect on Mg^2+^-driven activity. This inhibition exhibited a dose-dependent pattern (Fig 4F) with an estimated IC_50_ of 205.7 ± 2.8 μM (Fig 4F—*inset*).

### Intracellular localization of *Tc*GS

**Fig 5:**
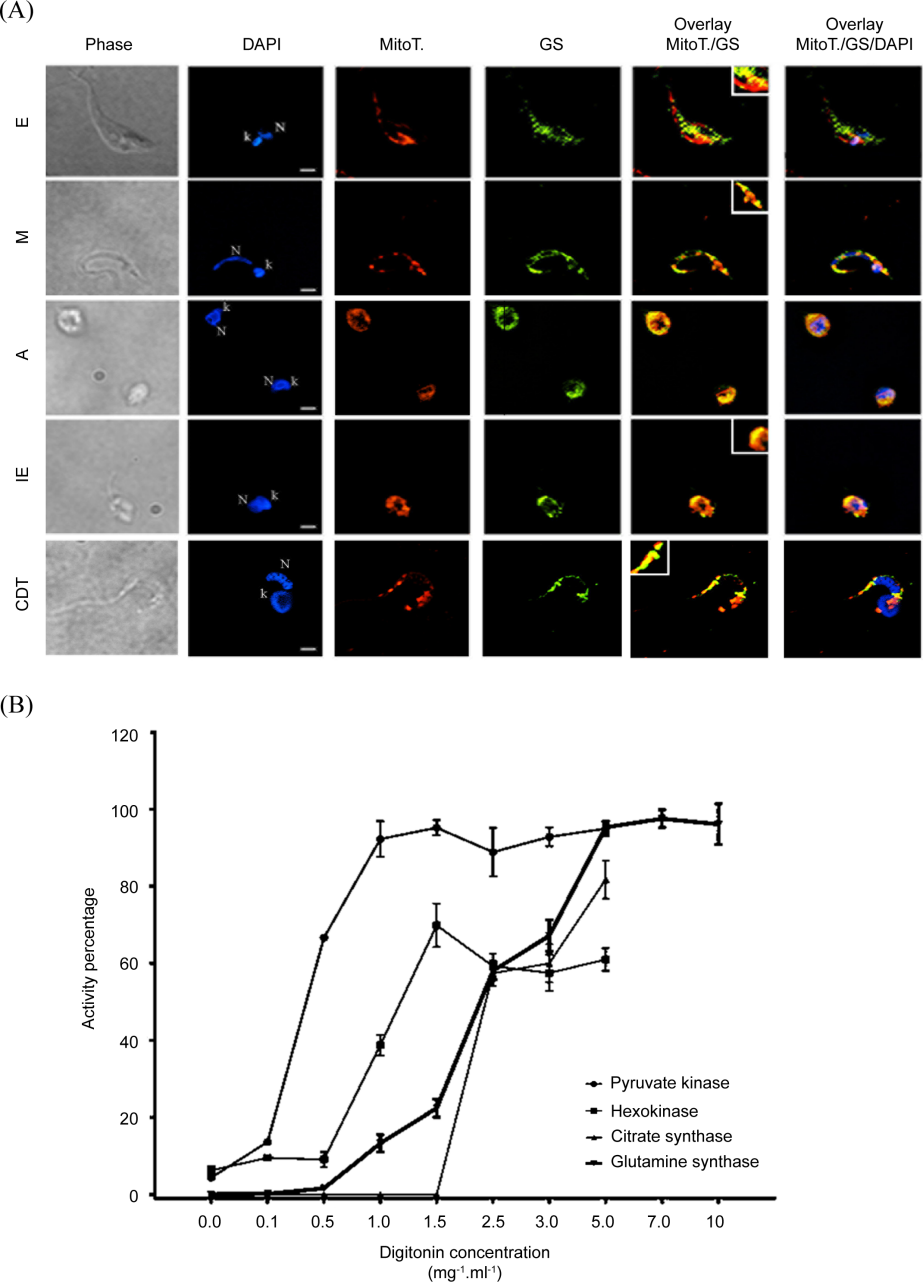
Subcellular localization of GS in *T*. *cruzi*. (A) All stages of *T*. *cruzi* life cycle (E–Epimastigote; MT–Metacyclic trypomastigote; A–Amastigote; IE–Intracellular epimastigote; and CDT–Trypomastigote) were harvested by centrifugation, immobilized on glass slides, incubated with anti-GS (green) and labeled with DAPI for DNA staining (blue) and MitoTracker Red Mito Sox for mitochondrial staining (red). Bars are 1 μm. N—nucleus, k–kinetoplast. (B) E were selectively permeabilized with increased digitonin concentrations (0–5 mg/ml), and the resulting supernatants (S) and pellets (P) were used for enzymatic assays. The enzymatic activities of pyruvate kinase (filled circles—cytosol marker), hexokinase (filled squares—glycosomal marker), citrate synthase (right-side up filled triangles—mitochondrial marker), and GS (upside-down filled triangles) were determined for all resulting fractions (S and P). The data correspond to the ratio between activities on S and P and were expressed as a percentage of the enzyme.

We performed immunofluorescence assays in all the parasite stages using an Anti-GS antibody (Sigma- Aldrich, St. Louis, Missouri) (S2) to determine the subcellular location of *Tc*GS and to extend these analyses to other life cycle forms. The enzyme was spread throughout the cytoplasm and inside the mitochondrial lumen in all life cycle forms (Fig 5A). We performed a differential permeabilization assay in E using digitonin in an attempt to confirm that the subcellular location indicated the presence of active enzyme. The permeabilization of different intracellular compartments was assessed by the release of marker enzyme activities into the medium: pyruvate kinase allowed us to trace the cytosolic fraction; hexokinase, the glycosome; and citrate synthase, the mitochondrial matrix. GS activity was found to be released in a two-step fashion. It was first observed at digitonin concentrations higher than those needed to release pyruvate kinase but smaller than those necessary to release mitochondrial citrate synthase. However, when the digitonin concentration was high enough to release citrate synthase, glutamine synthetase activity increased significantly (Fig 5B). Taken together, both sets of data strongly support a dual (cytoplasmic and mitochondrial) localization of the active enzyme.

### Expression levels and activity pattern of glutamine synthetase on the life cycle stages of *T*. *cruzi*

**Fig 6:**
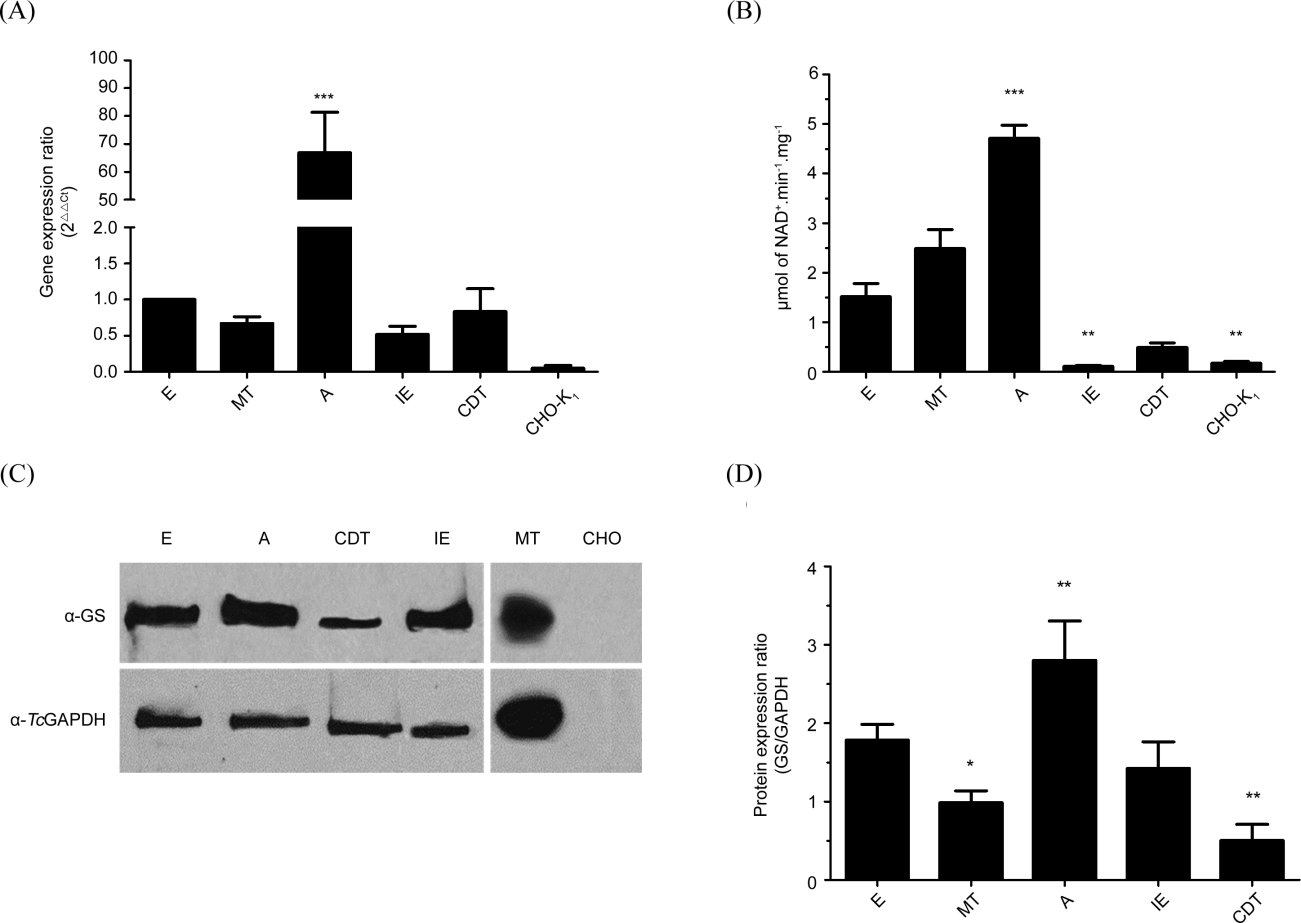
Expression profile of GS in *T*. *cruzi*. (A) Quantification of transcripts for the *Tc*GS gene in the different stages of *T*. *cruzi*. The mRNA was quantified by qRT-PCR using primers for a fragment of the gene. The *Tc*GAPDH gene was used as a normalizer (kept in-house). The expression ratio was calculated by the 2^-ΔΔCt^ method, in which E stage expression was defined as 1. The cDNA of CHO-K_1_ cells was used as a negative control since this strain is used to obtain the intracellular forms of *T*. *cruzi*. The differences between the expression profiles were evaluated by a one-way ANOVA test (p <0.05). In (B), the specific activity of GS was evaluated in the different stages of *T*. *cruzi*. The measurement of enzymatic activity was performed as described previously for the parasite extracts. (C) shows a representative Western blot performed with protein extracts of the *T*. *cruzi* stages. The extracts were resolved on denaturing polyacrylamide gel (10% acrylamide), transferred to a nitrocellulose membrane and incubated with anti-GS antibody (Sigma- Aldrich, St. Louis, Missouri, USA). The membranes were incubated with a secondary anti-rabbit IgG (Sigma- Aldrich, St. Louis, Missouri, USA) antibody for chemiluminescent detection. In this case, the stripping process was performed by adding 0.2 M NaOH. After two washes with PBS, incubation was performed using the same process described above, but the primary antibody was changed to anti-GAPDH (Sigma- Aldrich, St. Louis, Missouri, USA). Lanes: E: Epimastigote; A: Amastigote; CDT: Trypomastigotes; IE: Intracellular epimastigotes; MT: Metacyclic trypomastigotes; CHO: non-infected CHO-K_1_ cells. (D) Densitometry analysis of Western blot bands (three independent experiments including that of panel C). Asterisks indicate statistical analysis by 1way ANOVA / Tukey’s Multiple Comparision Test for (A) and Dunnet’s Multiple Comparision Test for (B) and (D); *p<0.05, **p<0.01, ***p<0.001, error bars represent standard deviation (n = 3).

Expression of the *TcGS* gene was analyzed by qRT-PCR in all five *T*. *cruzi* stages. mRNA levels were higher in A than in E, showing a dramatic ca. 70-fold increase (Fig 6A). In contrast, the other forms displayed modest differences in mRNA levels compared with those of E. GS activity was also measured. In agreement with the gene expression data, GS activity was higher in the A form of the parasite, albeit it was ca. 5-fold greater than that measured in E. In addition, the E and MT forms showed significant GS activities (Fig 6B). These all contrasted with the IE and CDT forms, where only near background activity levels were observed. Protein levels were evaluated by Western blot (Fig 6C and 6D). The A form GS levels were again the highest among all life forms of *T*. *cruzi*. However, the profile was less sharp than it was in the other two analyses, and this life form showed only a ca. 1.4-fold greater amount of protein compared with the E form. Finally, CDT showed the smallest amount of *Tc*GS protein, which is similar to that observed for mRNA expression and GS activity in this life form.

### The effects of inhibiting GS

**Fig 7:**
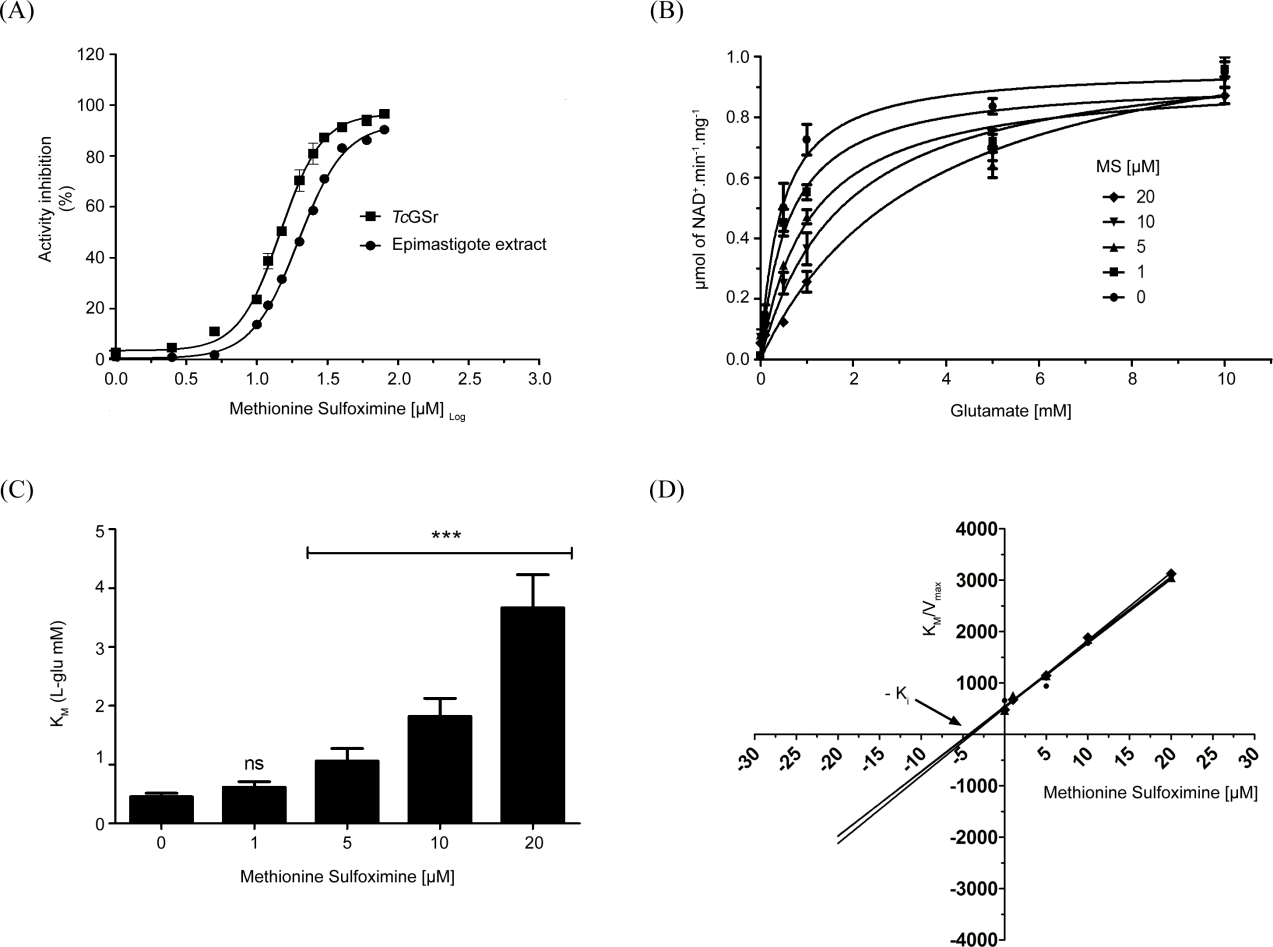
MS as a GS inhibitor. (A) The extract of epimastigotes (filled circles) and the recombinant enzyme *Tc*GS (filled squares) were both incubated with different MS concentrations for 30 min. Then, the specific activity was measured and the inhibition percentage was calculated. The values were adjusted to a dose-response curve (IC_50_ for cell-free extracts: 19.66 ± 0.37 μM, IC_50_ for *Tc*GS: 14.70 ± 0.54 μM). The data refer to three independent experiments. (B) GS activity was measured with different MS concentrations for 30 min; at the same time, it was measured in different glutamate concentrations. The data were adjusted to a Michaelis-Menten equation. (C) The K_M_ values for each MS concentration were compared. (D) The relationship between K_M_/V_max_ per MS concentration revealed the inhibitory constant value (K_i_ = 4.12 ± 0.21). Statistical analysis were made using one-way ANOVA / Dunnet’a Multiple Comparision Test. *p<0.05, **p<0.01, ***p<0.001. Error bars represent standard deviation (n = 3).

Inhhibiting GS activity in the parasite was necessary to unveil the biological roles of GS. As TcGS are described as essential enzymes in most of organisms, and there are no available inducible knock down or knock out methods for essential genes in *T*. *cruzi*, we used a well-known chemical inhibitor considered specific for the enzyme, Methionine sulfoximine (MS) [45]. Recombinant *Tc*GS activity (expressed in *E*. *coli*) and GS activity from E cell-free extracts were susceptible to MS in a dose-dependent manner. Their IC_50_ values were similar and estimated to be 20.72 ± 0.07 μM and 38.85 ± 0.08 μM, respectively, for recombinant *Tc*GS and GS (Fig 7A). Kinetic analysis results showed that MS changes the Michaelis-Menten pattern of GS (Fig 7B), maintaining its V_max_ value but increasing the K_M_ values, by acting as a competitive inhibitor with respect to L-glutamate (Fig 7C); the K_i_ values were estimated to be 3.89 ± 0.04 μM and 4.65 ± 0.08 μM for the recombinant enzyme and GS activity in E cell extracts, respectively (Fig 7D).

**Fig 8:**
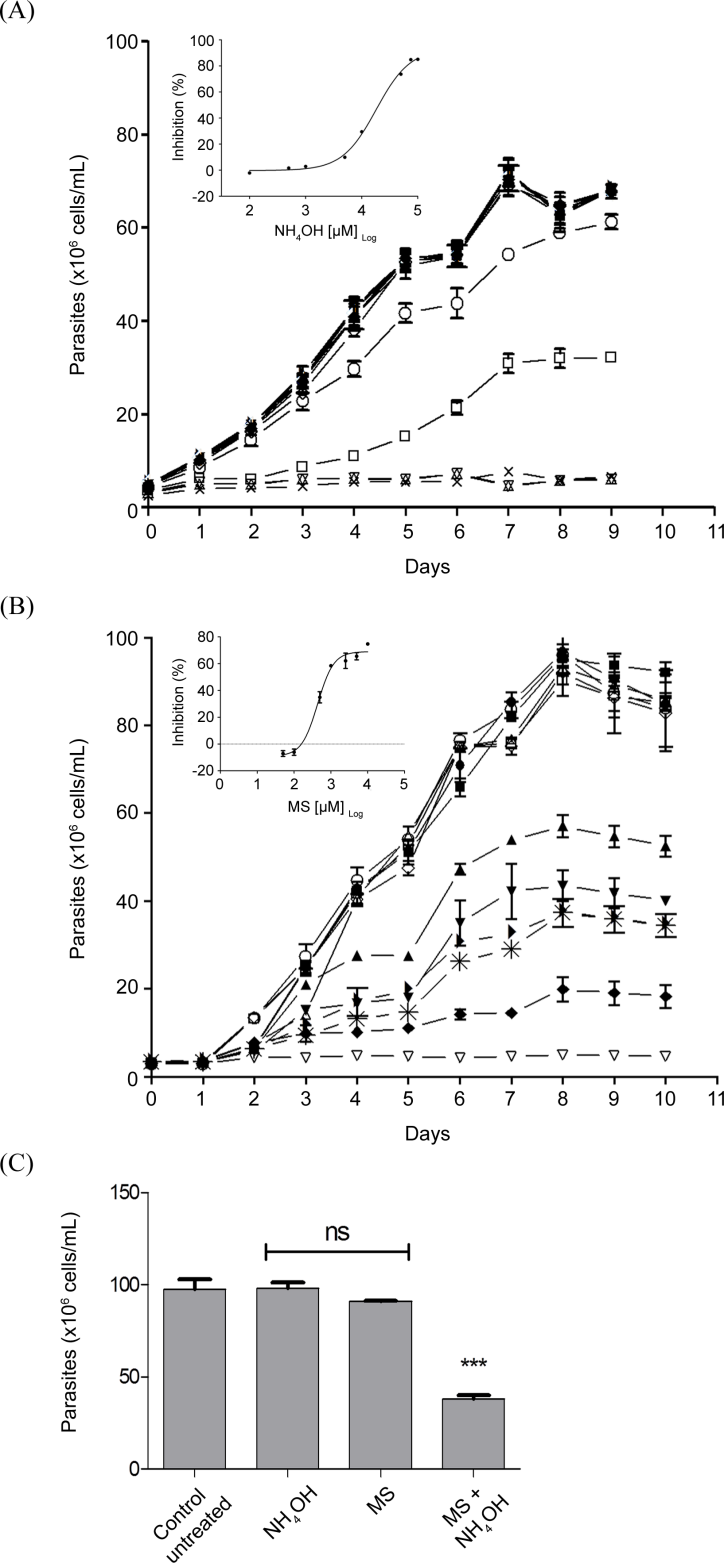
Effect of extracellular NH_4_^+^, MS and their combination on E proliferation. (A) The effect of different concentrations of NH_4_^+^ on E replication was evaluated by culturing them in liver infusion tryptose (LIT) medium at 28°C supplemented with different concentrations of NH_4_OH (in all cases, the pH was adjusted to 7.4). Parasites were quantified daily as previously described [46]. Symbols: filled circles: 1 μM; filled squares: 5 μM; right-side up filled triangles: 10 μM; upside-down filled triangles: 50 μM; right-side up filled triangles: 100 μM; left filled triangles: 500 μM; filled diamonds: 1 mM; open diamonds: 5 mM; open circles: 10 mM; open squares: 50 mM; right-side up open triangles: 75 mM; right open triangles: 100 mM; asterisks: untreated (control); x symbol: 0.5 μM antimycin and 60 μM rotenone (100% growth inhibition control). Inset: dose-response curve (EC_50_ = 17.77 ± 0.99 mM). (B) The effect of different concentrations of MS and 5 mM NH_4_OH on E replication was evaluated by culturing them in LIT medium at 28°C. Parasites were quantified daily. Symbols: filled circles: 0.05 mM; filled squares: 0.1 mM; right-side up filled triangles: 0.5 mM; upside-down filled triangles: 1 mM; right filled triangles: 2.5 mM; asterisks: 5 mM; filled diamond: 10 mM; open diamonds: 5 mM MS without NH_4_OH; open circles: 10 mM MS without NH_4_OH; open squares: control (no treatment); right-side up open triangles: 5 mM NH_4_OH without MS; upside-down open triangles: 0.5 μM antimycin and 60 μM rotenone (100% growth inhibition control). Inset: dose-response curve for MS in the presence of NH_4_OH (EC_50_ = 438.4 ± 47.4 μM). (C) The combined effect of 1 mM MS and 5 mM NH_4_OH on epimastigote proliferation measured at the mid-exponential phase of untreated cultures (4th day of proliferation). Statistical analysis were made using one-way ANOVA / Dunnet’a Multiple Comparision Test. *p<0.05, **p<0.01, ***p<0.001. Error bars represent standard deviation (n = 3).

Once we characterized the inhibition of *Tc*GS by MS on the enzyme, we were interested in evaluating its effect on the parasite. Thus, we initially cultured E in the presence of different concentrations of MS. As previously shown, MS had a limited effect at concentrations up to 1 mM, showing an IC_50_ concentration of 17.0 ± 0.3 mM [42]. However, we evaluated the interaction between significant but non-lethal levels of ammonium and MS to account for our initial hypothesis that GS would be involved in NH_4_^+^ management. In these conditions, MS was able to inhibit parasite proliferation with a dose-dependent profile. The EC_50_ was 438.4 ± 47.4 μM, showing a nearly 40-fold increase in parasite sensitivity to MS (Fig 8A–8C), which reciprocally means that the specific inhibition of *Tc*GS also increases the sensitivity of *T*. *cruzi* E to the presence of NH_4_^+^.

**Fig 9:**
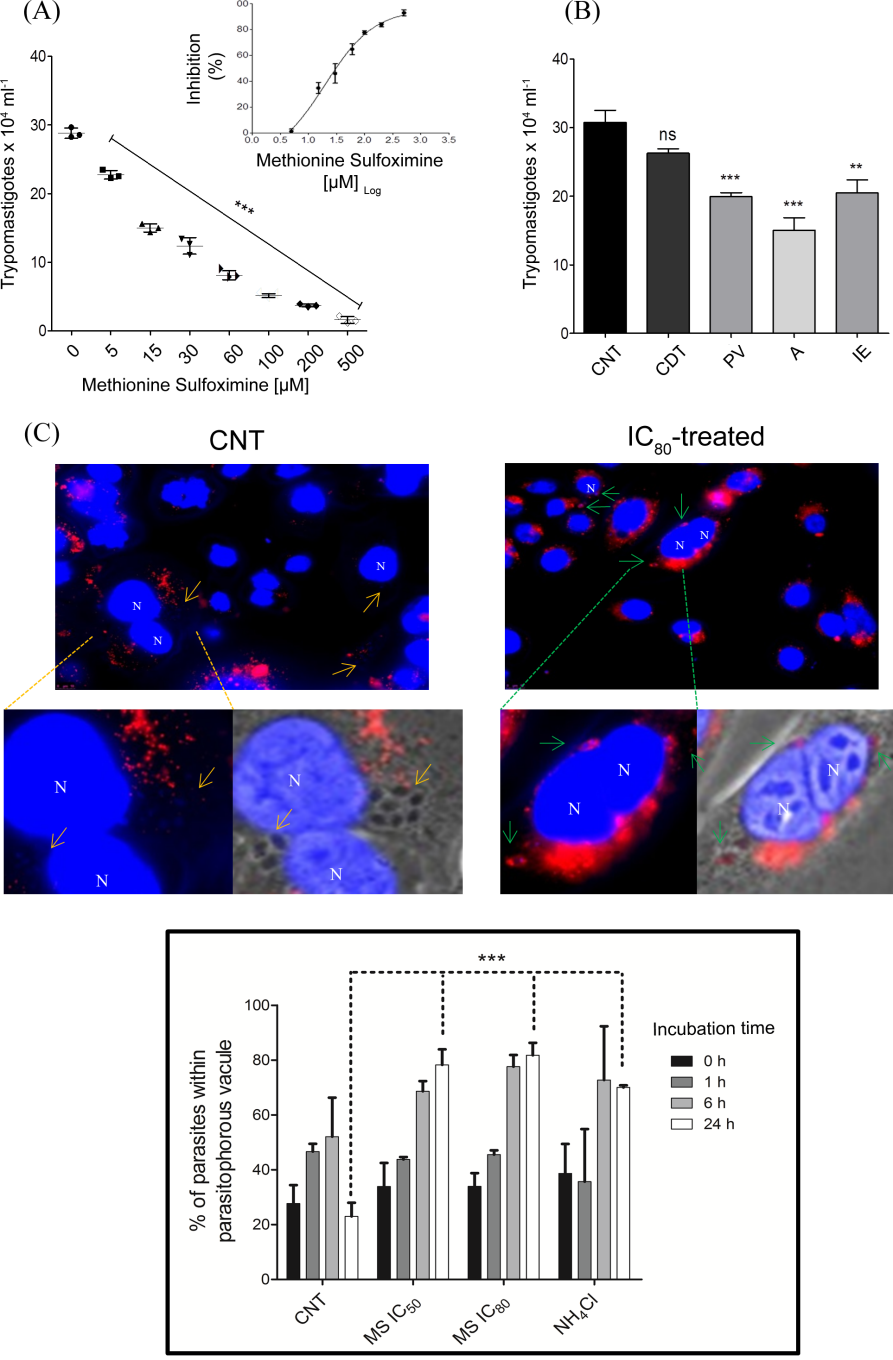
Effect of MS on the intracellular cycle of *T*. *cruzi*. (A) The effect of MS on CDT bursting was evaluated by treating the cells throughout the entire infection cycle (concentrations varied between 5 and 500 μM). CDT forms were counted in the culture media at day 5 post-infection. Inset: dose-response curve for the effect of MS treatment on CDT bursting (EC_50_ = 20.02 ± 0.91 μM). (B) Effect of MS on the different moments of intracellular infection. The mammalian host cells were infected under synchrony conditions in such a way that all infected cells were displaying the same intracellular stages at any moment, as previously described [15]. The infected cells were treated with 20 μM MS (dose corresponding to the EC_50_) for 3 h during the invasion, for 24 h immediately after invasion (in our system, this time period is when parasites remain inside the parasitophorous vacuole—PV), between 24 and 48 h (intracellular parasites are already in A form in the host-cell cytoplasm) or between 48 and 120 h post-infection (intracellular parasites are in IE form). The measured effect was CDT bursting, which was quantified by counting parasites in a Neubauer chamber. (C) Representative images of the PV of cells treated (or not, as a control) with MS. The assays were performed by labeling CHO-K_1_ infected cells with LysoNIR. After 3 h of infection the cultures were submitted to a 24-h treatment (or not) with 83 μM MS (corresponding to EC_80_) and washed. Nuclear DNA (N) was stained with Hoechst 33342. *Yellow arrows*: example of unlabeled parasites; *green arrows*: example of labeled parasite*s*. *Bottom*: the total number of parasites and number of parasites labeled with LysoNIR 200 cells were counted and used to calculate the percentage of parasites within the PV at different times post-infection. Statistical analysis were made using one-way ANOVA / Dunnet’a Multiple Comparision Test. *p<0.05, **p<0.01, ***p<0.001, Error bars represent standard deviation (n = 3).

During the mammalian host-cell infection (particularly in the intracellular environment), the parasites undergo to a metabolic switch, consuming mainly proline instead of glucose [16]. Thus, as *Tc*GS is relevant for E to address NH_4_^+^ toxicity, it could predictably be relevant to managing NH_4_^+^ toxicity during host-cell infection by *T*. *cruzi*. In this regard, CHO-K_1_ cells were infected with CDT, treated with different concentrations of MS (or not treated, as a control) throughout the entire intracellular cycle, and the number of burst CDT was recorded. MS decreased the number of released CDT in a dose-dependent manner compared to the control (Fig 9A). If the production of CDT is taken as a measurement of the effect of MS, an EC_50_ of 20.02 ± 7.88 μM could be calculated (Fig 9A *inset*). Whole host-cell infection is a complex process, involving several steps of replication and differentiation. Thus, the question of whether the treatment of infected CHO-K_1_ cells was affecting all the intracellular stages and their differentiation processes remained unanswered. We explored this point with synchronized infections as previously described [15] and treated the infected cells at defined time-points to measure the effect of MS on: i. the invasion process (first 3 h before culture wash); ii. the parasite survival in the parasitophorous vacuole and/or further exit of the parasitophorous vacuole; and iii. the effect of inhibiting *Tc*GS on the intracellular stages A and IE. Our results show that A proliferation was inhibited to a higher extent than IE (51% and 34%, respectively) (Fig 9B). Interestingly, when the infected cells were treated with MS for the first 24 h post-infection, which initiated exposure to the drug immediately after invasion, a significant reduction in the number of released CDT was observed, raising the question of whether the inhibition of *Tc*GS could cause the parasite to fail to escape from the vacuole. We showed that such inhibition of *Tc*GS impaired the evasion of the parasitophorous vacuole (Figs 9C and S3). These results implicate *Tc*GS as the first metabolic enzyme involved in *T*. *cruzi* evasion from the parasitophorous vacuole.

## Discussion

Despite being a principal enzyme in amino acid and nitrogen metabolism in all studied living organisms, the GS of parasites has received little attention. Its presence and activity were previously shown in *T*. *cruzi* [41,42] and *L*. *donovani* [40], but deep biochemical and functional characterizations have been lacking. In this work, we show such characterizations in a GS encoded in the *T*. *cruzi* genome. In addition, we show the involvement of TcGS in managing the stress caused by an accumulation of NH_4_^+^ in the extracellular environment using the specific GS inhibitor MS. Finally, we show the involvement of *Tc*GS in the evasion of *T*. *cruzi* from the parasitophorous vacuole, which is a critical step for the parasite to initiate intracellular replication and thus establish infection. To the best of our knowledge, this report is the first to link the activity of a metabolic enzyme to the ability of a parasite to reach its intracellular niche in order to replicate and establish cell infection.

### Biochemical aspects of *Tc*GS and subcellular location

We initially confirmed the GS activity of the *Tc*GS gene found in the *T*. *cruzi* databases through a functional yeast complementation assay. Once we confirmed that *Tc*GS encodes a functional GS, we were interested in performing a biochemical characterization by obtaining an active recombinant enzyme expressed in *E*. *coli*. While this enzyme conforms to a canonical GS in many aspects, it shows some important differences. Eukaryotic GSII enzymes have been described to form oligomers composed of 8–10 monomers, with the latter organization being more frequent in the enzymes of plant origin [29]. In this respect, the size-exclusion data for *Tc*GS agree with oligomeric organization as an octamer, which resembles the conformation found in mammals and fungi [38,47]. In terms of substrate specificity, *Tc*GS activity was dependent on L-glutamate, NH_4_^+^ and Mg·ATP. Interestingly, L-glutamate analogs could support its activity to some extent, but no other tested amino acid was effective beyond 10% of the activity found with L-glutamate. These data depict an enzyme that is similar to its corresponding plant and animal enzymes [48]. In relation to co-factors, Mn^2+^ is the preferred cation for activity in bacterial GSs, but it is less effective than Mg^2+^ in eukaryotic GSs (e.g., [33,39]). Nevertheless, Mn^2+^ may be an important factor in regulating GS activity in eukaryotes; for example, the concentration of Mn^2+^ is close to the K_d_ concentration in the cytosol of brain cells, and it varies as a response to Ca^2+^ signaling, which likely affects GS activity [49]. *Tc*GS shows a behavior similar to that found in eukaryotic GS: Mg^2+^ is the preferred cation, albeit Mn^2+^ (and to a lesser extent, Co^2+^) can serve as a substitute.

GSs are cytosolic enzymes in most studied organisms. However, additional mitochondrial locations have been well established for uricotelic vertebrates [31,50], higher plants [51] and *Drosophila melanogaster* [37]. These dual localizations have been explained by the existence of weak mitochondrial-location determinants in the *N*-terminus domains of these proteins in the two former cases or by the existence of two different isoforms in the latter case [51,52]. In this context, it was important but not surprising that *Tc*GS was observed to be a dual-location enzyme with a presence in both the cytosol and mitochondria. In fact, this result is consistent with the fact that the first step of amino acid metabolism, which consists of amino acid deamination or transamination, mostly occurs in one or both of these locations. Furthermore, the main enzymes involved in these processes and in NH_4_^+^ management (i.e., tyrosine and aspartate aminotransferases and glutamate dehydrogenase) [22–25,53,54] also have mitochondrial and cytosolic localization. Furthermore, the present results suggest that localization to both the cytosol and mitochondria occur constitutively since this localization pattern does not seem to change during the different life stages of the parasite. Taken together, this information allowed us to propose that *Tc*GS is a constitutive part of the NH_4_^+^ detoxification system in *T*. *cruzi*.

Mitochondria are organelles displaying high-capacity Ca^2+^ transport systems, and consequently, they store substantial amounts of this cation, mostly in the form of Ca^2+^ phosphates [55]. Trypanosomatid mitochondrion are not an exception: they show vigorous Ca^2+^ transport systems akin to those found in mammalian mitochondria [56]. Even though this cation mostly accumulates in an inert form, mitochondria typically show concentrations of free Ca^2+^ that are nearly two orders of magnitude greater than those in the cytoplasm under non-stimulated conditions, i.e., 1–5 μM [55]. In this regard, Ca^2+^ sensitivity by GS was observed in early research on eukaryotic enzymes [57,58]. In the case of mitochondrial GS, this inhibition may play a role in regulating its activity in vivo. However, *Tc*GS shows negligible inhibition in the low micromolar range. Conversely, its half-maximal inhibition was found to occur at a Ca^2+^ concentration two orders of magnitude greater than its mitochondrial concentration. On the other hand, Ca^2+^ inhibition cannot be ascribed to ATP sequestration because at concentrations near the IC_50_ (e.g., 0.2 mM), [Mg·ATP] in the reaction mixture is predicted to be ca. 0.73 mM, whereas [Ca·ATP] is only ca. 0.11 mM [59]; these figures are comparable to a predicted [Mg·ATP] ≈ 0.73 mM in the absence of Ca^2+^. With all this in mind, it can be concluded that the effect of Ca^2+^ on *Tc*GS is that of a *bona fide* inhibitor, but the physiological implications of this inhibition are unclear.

### The role of *Tc*GS in the biology of *T*. *cruzi*

We were initially interested in evaluating the possible regulation of *Tc*GS during the parasite’s life cycle to infer (and then demonstrate) the possible role(s) of *Tc*GS in *T*. *cruzi* (beyond the obvious role of supplying glutamine for protein synthesis). When *Tc*GS was evaluated in terms of gene expression, protein amounts, or GS activity in the in vitro forms representing all the stages of the natural life cycle of the parasite, we found that the intracellular stage amastigote of *T*. *cruzi* showed higher amounts of GS-encoding mRNA, protein and enzyme activity. This expression pattern correlates with a metabolic switch from a life stage in which glycolysis prevails (CDT) to another in which amino acid metabolism is prevalent (A) [15][16]. These observations reinforced the hypothesis of a role of *Tc*GS in the management of excess NH_4_^+^ produced by *T*. *cruzi* metabolism under these conditions. Corroboration of this idea requires a reliable and feasible method to diminish *Tc*GS activity. As *Tc*GS is described as a central enzyme in the metabolism of most studied organisms [45], we chose to chemically inhibit it using the chemical inhibitor MS [60]. *Tc*GS sensitivity to MS was found to fall in the micromolar range. This result is in sharp contrast to the millimolar values found for the human enzyme [60], and at first sight, it is seen an opportunity for intervention against the parasite because GS activity is involved in glutamine synthesis [61,62]. Therefore, it was somewhat surprising to observe that MS had little effect on the proliferation of E even though *Tc*GS showed remarkable sensitivity to this compound. However, it must be noted that during replication, the metabolism of the E form is mainly based on glucose consumption, thus producing little amounts (if any) of NH_4_^+^. This consumption is the reason for minimal amounts of GS at this stage, both in terms of its activity and mRNA level. The sensitivity of E replication to MS was evaluated in the present of different concentrations of NH_4_OH supplemented to the culture medium to confirm that the effect of MS was related to the accumulation of extracellular NH_4_^+^. MS affected E replication when NH_4_^+^ was present in millimolar amounts in the growth medium; under these conditions, proliferation was severely halted by concentrations of MS that had no previous effect, showing the involvement of *Tc*GS in NH_4_^+^ detoxification. Summarizing, *Tc*GS is involved in the management of NH_4_^+^ accumulation by incorporating it on glutamate, which in turn can be obtained by the amination of α-ketoglutarate by glutamate dehydrogenases [23–25] or transaminases [22], by the oxidation of proline[63,64] or by its uptake from the extracellular medium[65].

Once we established the role of *Tc*GS in the management of NH_4_^+^ production, it became evident that there was a relevant role for this enzyme in the intracellular life cycle stages, which are dependent on amino acid metabolism. First, we observed that the bursting of CDT forms from infected CHO-K_1_ diminished with MS treatment in a dose-dependent manner, showing that the inhibitor was affecting at least one process during the parasite’s intracellular cycle. After host-cell invasion, the CDT are initially found in vacuoles that undergo lysosome fusion to hold the parasite inside (parasitophorous vacuoles) [66–69]. Once inside these vacuoles, the invading trypomastigotes (MT or CDT) initiate a differentiation process to A, which is able to evade the vacuole into the cytoplasm in an acidic-pH-dependent way [7–10,70] to initiate intracellular proliferation in the cytosol [9,67,68,71–73]. Notably, Ley et al. previously showed that *T*. *cruzi* fails to escape from the parasitophorous vacuole when it is alkalinized by NH_4_Cl [9]. In this work, the inhibition of GS activity impaired parasitic vacuole evasion by A, the parasite stage displaying the highest GS activity. Interestingly, this process was also affected in a dose-dependent manner, and notably, MS showed its effect on CDT production at concentrations similar to the estimated IC_50_ of the recombinant enzyme. Remarkably, CHO-K_1_ is almost 10^3^ times less sensitive to MS (IC_50_ > 20 mM, selectivity index > 849.15) [42], indicating that the observed phenomenon was not due to a toxic effect of MS on the host cells.

**Fig 10:**
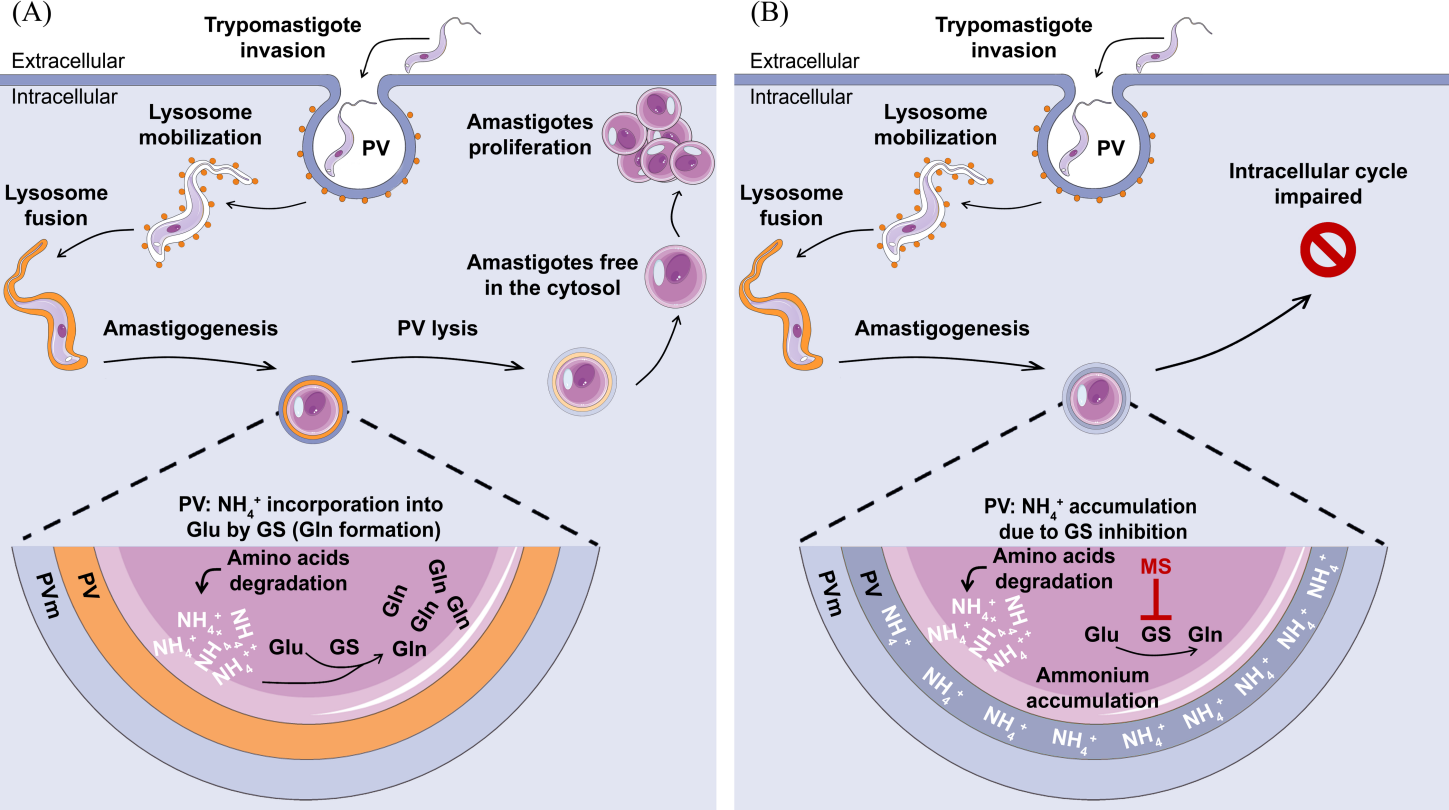
Schematic proposal for the role of *Tc*GS in the progression of intracellular infection by *T*. *cruzi*. CDT forms invade the mammalian host cells by forming a parasitophorous vacuole, which involves the recruitment of lysosomes to fuse the vacuole containing the parasite. The lysosome fusion event triggers the acidification of the vacuole and promotes amastigogenesis [7,9,74]. It is well established that A are amino acid consumers; thus, ammonium production (which should be managed by the metabolic network involving glutamate dehydrogenases, aspartate aminotransferases and *Tc*GS) is expected (Panel A). We propose that if *Tc*GS is inhibited, then the production of ammonium overloads the transamination system, and an impairment of PV evasion occurs. Furthermore, if GS is inhibited after PV evasion and during A proliferation, infection is also impaired (Panel B).

In summary, considering the following:

1. *T*. *cruzi* forms a very tight parasitophorous vacuole (indicating that the production of little amounts of any metabolite would give rise to significant concentrations);
2. after its differentiation into A inside the vacuole, amino acid metabolism starts with the production of NH_3_;
3. the acidic medium in the vacuole favors the spontaneous conversion of NH_3_ into NH_4_^+^, which quickly increases intra-vacuolar pH; and
4. parasite evasion from the vacuole depends on enzymatic activities that are triggered at acidic pH [7–10,70],

we propose that *Tc*GS is involved in the maintenance of intracellular pH by contributing to the regulation of the intravacuolar content of NH_4_^+^ (Fig 10). We also show that interference with this system affects the efficiency of infection. To our knowledge, no reports on off-target effects of MS were published so far. However, this possibility cannot be ruled out. Upon the availability of methods allowing to perform knock-out or knock-down of essential genes in an inducible way in amastigotes, the involvement of GS in the evasion of parasites from the PV into the cytoplasm during infection of host-cells will reinforce the findings reported herein.

## Materials and methods

### Cells and parasites

E from CL strain clone 14 were maintained in exponential growth phase by subculturing every 48 h in LIT medium supplemented with 10% FCS at 28°C as previously described [75]. The Chinese Hamster Ovary cell line CHO-K_1_ (kindly provided by Maria Júlia Manso Alves, Department of Biochemistry, Institute of Chemistry, University of São Paulo, São Paulo—Brazil) was cultivated in RPMI medium supplemented with 10% heat-inactivated fetal calf serum (FCS), 0.15% (w/v) NaCO_3_, 100 units/ml penicillin and 100 μg/ml streptomycin at 37°C in a humid atmosphere containing 5% CO_2_. E of *T*. *cruzi* CL strain clone 14 [76] were maintained in the exponential growth phase by subculturing every 48 h in LIT medium supplemented with 10% FCS at 28°C. CDT were obtained by infection of CHO-K_1_ cells as described previously [46].

### Yeast strains, plasmids and growth conditions

Cells were routinely grown on standard YP or YNB-based drop-out media supplemented with appropriate carbon sources. Introduction of plasmids into yeast cells was done by the lithium-acetate method [77]. The SAH35 strain is a derivative of W303-1a (MATa *leu2-3*,*112 trp1-1 can1-100 ura3-1 ade2-1 his3-11*,*15 GLN1*UAS::*GAL1*UAS-6xHis*GLN1*-NATr) in which the endogenous upstream 5’ untranslated and promoter sequences of the gene *GLN1* were substituted with those from the *GAL1* gene using a linear DNA construct and one-step gene replacement procedures. Briefly, plasmid pAH-GG1 was constructed by introducing PCR-amplified genomic sequences comprising nucleotides -569 to -246 with respect to *GLN1* translation start and the ORF of GLN1 as *Hind*III-*Bgl*II and *Bam*HI-*Cla*I fragments into plasmid pAH-N15, respectively. The latter is a modified version of pYM-N15 [78] obtained from EUROSCARF (http://www.euroscarf.de/index.html) that had its *GPD1* promoter substituted with a *GAL1* promoter. In addition to the genomic sequence, the *GS* ORF fragment also included a His_6_ tag to be expressed as an *N*-terminus domain. The 2746 bp long HindIII-EcoRI fragment obtained from pAH-GG1 was introduced into W303-1a, and transformants were selected on YPGal plates supplemented with 0.1 mg/ml nourseothricin. Complementation assays were done by drop tests, as previously described [79], on YNB-based drop-out media substituting (NH_3_)_2_SO_4_ with 10 mM glutamate as a non-repressible *N*-source and, where indicated, supplementing with 100 mg/l glutamine [80].

### Cloning, expression and purification of the recombinant TcGS protein

The putative *Tc*GS gene (TcCLB.503405.10—template sequence) was identified from the *T*. *cruzi* genome project database (http://www.genedb.org). The *Tc*GS coding region was amplified by PCR using *T*. *cruzi* CL14 strain genomic DNA as a template and gene-specific primers designed with restriction sites for the enzymes *Bam*HI and *Xho*I: *Tc*GS forward 5′-AAGGATCCATGACAGGC TTGAAGGAGAAAAG -3′ and *Tc*GS reverse 5′-GGCTCGAGTGACAAATCGCCAAATTTCATCC -3′. PCR amplification settings were set at 95°C (5 min) and 32 cycles using the following conditions: initial denaturation cycle at 92°C (1 min), annealing at 60°C (1 min) and elongation at 72°C (2 min). A single fragment (1.043 kb) was amplified and the PCR product was purified and cloned into the pGEM-T Easy vector (Promega, Madison, WI, United States). Selected clones were sequenced, and the expected identity of the cloned DNA fragments to GS was confirmed using the BLAST software program (http://blast.ncbi.nlm.nih.gov/). The gene encoding the putative GS enzyme was further subcloned into the pET28a(+) expression vector as previously described [34], and the construct was used to transform *E*. *coli* BL21-CodonPlus (DE3) cells. The bacteria were grown in Luria-Bertani (LB) medium containing 100 μg/ml Kanamycin and 5 μg/ml Tetracycline at 37°C until an OD_600_ of 0.6 was reached. Expression of *Tc*GS was induced by the addition of the enzyme substrates (100 μM) and isopropyl-1-thio-β-D-galactopyranoside (IPTG) to a final concentration of 0.25 mM, and cells were maintained at 16°C for 24 h. For protein purification, the cells were harvested, resuspended in lysis buffer (50 mM Tris-HCL pH 7.5, 500 mM NaCl, and 1 mg/ml lysozyme) containing protease inhibitors and subjected to 10 cycles of sonication (ten 30 sec pulses followed by 30 sec of rest between cycles). The recombinant His_6_-tagged protein was purified using Ni^2+^ nitrilotriacetic (NTA) column affinity chromatography (Qiagen, Hilden, North Rhine-Westphalia Germany) according to the manufacturer’s instructions.

### Reverse transcription PCR (RT-PCR) and quantitative real-time PCR (qRT-PCR)

Total RNA was extracted from different *T*. *cruzi* stages and CHO-K_1_ cells (control) using TRIzol reagent (Invitrogen, Life Technologies, Carlsbad, California, United States). RNA preparations were treated with RNase-free DNase I (Fermentas, Life Sciences, Waltham, Massachusetts, United States) and checked by running aliquots in 1% agarose gels. Reverse transcription was performed with SuperScript IITM (Invitrogen, Life Technologies, Carlsbad, California, United States) using the anti-sense Oligo (dT) primer, 5 μg of RNA and by following the manufacturer’s instructions. The primers used for qRT-PCR analysis were designed using software PrimerBlast (NCBI). Primers were designed based on the nucleotide sequences of *T*. *cruzi* glyceraldehyde-3-phosphate dehydrogenase (GAPDH) (GenBank accession number: AI007393), which was used as a housekeeping gene [27], and *Tc*GS (GenBank accession number–template sequence: XP_803102.1). The primer sequences were GAPDH forward (5′-GTGGCAGCACCGGTAACG-3′), GAPDH reverse (5′-CAGGTCTTTCTTTTGCGAAT-3′), *Tc*GS forward (5′- AAGGATCCATGACAGGCTTGAAGGAGAAA AG -3′) and *Tc*GS reverse (5′- GGCTCGAGTGACAAATCGCCAAATTTCATCC-3′). qRT-PCR analyses were performed using Mastercycler ep REALPLEX 1.5 (Eppendorf, Hamburg, Germany) equipment and a SYBR Green QuantiMix EASY SYG KIT (Biotools Quantimix EasySyg, Madrid, Spain) for amplicon quantification. PCR conditions were as follows: initial denaturation at 95°C (10 min) followed by 40 cycles of 94°C (1 min), 62°C (1 min) and 72°C (2 min). In all cases, denaturation curves for the PCR products were obtained. Data obtained were analyzed using REALPLEX v1.5 software. A fold-change in the expression of transcripts was obtained using the 2^-ΔCT^ method [81]. All time-fold variations were calculated using GAPDH as a housekeeping gene. cDNA from CHO-K_1_ cells was used as a control.

### Protein extracts of *T*. *cruzi*

The protein extracts were obtained from cells in exponential growth phase (5 x 10^7^ cells/ml). Cells were harvested by centrifugation, washed 3 times with PBS and resuspended in TSB buffer. The parasites were lysed by sonication with 5 cycles of 30 sec with 30-sec rest intervals between cycles. After centrifugation at 12,000 x *g* for 30 min, the supernatants containing the protein extracts were used in enzyme activity assays and Western blotting. Quantitation of total protein was done by the classical method of Bradford [82] using a solution of BSA (bovine serum albumin) as a standard to construct calibration curves.

### SDS-PAGE and western blotting

Sodium dodecyl sulfate polyacrylamide gel electrophoresis (SDS-PAGE) was undertaken using 10% (v/v) polyacrylamide gels according to the method described in [83]. Briefly, the protein extracts (Supernatants corresponding to 2x10^7^ *T*. *cruzi* cells or 5x10^6^ CHO-K_1_) or *Tc*GS recombinant (*Tc*GSr) was separated by (SDS-PAGE) and then transferred to nitrocellulose membranes (Supported Nitrocellulose Membrane, Bio-Rad, Hercules, California, U.S.A) using a Trans-Blot Semi-Dry Transfer Cell (Bio-Rad) [83]. After the transfer, the membranes were stained with Ponceau S (0.1% diluted in 10% acetic acid) and detained in tap water, allowing the evaluation of transfer efficiency. Membranes were blocked for 2 h with PBS-0.3% Tween20 (PBS-T) supplemented with 5% skim milk. After blocking, the membranes were incubated with primary antibody anti-GS (1:1000), stirring gently for 1 h at 4°C and were then washed three times for 5 min with PBS-T. Then, incubation was carried out for 45 min with secondary antibody conjugated with HRP enzyme (1:2500) (GE Healthcare, horseradish peroxidase), followed by washes as described above. We then performed detection using chemiluminescence reagent SuperSignal West Pico Chemiluminescent Substrate (Thermo Scientific, Waltham, Massachusetts, USA) according to the manufacturer's manual.

### Subcellular *Tc*GS localization

Exponentially growing E in different life cycle stages were resuspended in culture medium without serum containing 100 nM MitoTracker (Molecular Probes, Eugene, Oregon, United States) and treated according to manufacturer’s instructions for mitochondrial staining. Cells were washed with PBS and fixed with 4% (v/v) paraformaldehyde in PBS for 20 min at room temperature or with 100% methanol for 10 min. Paraformaldehyde-fixed cells were permeabilized with 0.1% Triton for 5 min. Fixed cells were washed three times with PBS and incubated with a 1:300 dilution of primary mouse antibody against GS for 1 h. The coverslips were rinsed three times with PBS and incubated with a 1:2000 dilution of goat anti-mouse IgG conjugated to Alexa Fluor 488TM in blocking solution for 1 h. After washing the coverslips three times in PBS, they were incubated with DAPI (1 mg/ml) and washed again with PBS. Images were acquired through a z-series of 0.2 μm using a lens of 100X 1.35NA with Cell R software in Olympus IX81 microscopy. Images were deconvoluted using Autoquant X2.1.

### GS activity assays

Two methods were used to determine the enzymatic activity of *Tc*GS. The first method was used in parasite extracts, and it measured NADH oxidation and the increase in absorbance at 340 nm. This molecule is substrate of lactate dehydrogenase (LDH).

Imidazole HCl buffer (34 mM), phosphoenolpyruvate (33 mM), magnesium chloride (2 mM), potassium chloride (18.9 mM) β-NADH (0.25 mM), pyruvate kinase (PK) (28 units) and LDH (40 units) were added to their respective concentrations in two quartz cuvettes (sample and white). In a first evaluation of the activity of GS, the substrates L-glutamate, NH_4_^+^ and ATP were adjusted to a final concentration of 1 mM. The measurements started with the recombinant enzyme or extract of *T*. *cruzi*, and a final oxidation of NADH at 340 nm was recorded. The reaction was started by adding 100 μg of the extract to the assay reaction mixture, which was incubated and measured for 10 min.

The second method determined *Tc*GS activity in the recombinant enzyme by measuring the production of inorganic phosphate (Pi) [84]. Before starting the reaction mix, the following solutions were prepared: **1.** Ammonium heptamolybdate solution: Approximately 32 ml of H_2_SO_4_ were carefully added into 100 ml of Milli-Q H_2_O in ice under laminar flow. Furthermore, we dissolved 3.7 grams of ammonium molybdate (Sigma- Aldrich, St. Louis, Missouri, USA) in 50 ml of Milli-Q H_2_O. The solutions were mixed, and 200 ml of Milli-Q H_2_O was added. The final solution was kept at room temperature and protected from light. **2.** Malachite green solution: 1 g of polyvinyl alcohol was dissolved in 50 ml of Milli-Q H_2_O. The solution was then filtered, and 18.5 mg of malachite green (Sigma- Aldrich, St. Louis, Missouri, USA) was added. The solution was mixed and stored at room temperature and protected from light.

K_M_ and V_max_ values for recombinant *Tc*GS were determined by regression analysis of the initial reaction velocity versus glutamate concentration using the Michaelis-Menten equation. The optimum pH for recombinant *Tc*GS activity was determined using a three-buffer system, which ranged from a pH of 5.0 to 9.0 and was composed of 34 mM each of MES, imidazole HCl or Tris Buffer. The kinetic parameters of the enzymatic reactions were calculated from at least three independent experiments.

Two methods were performed to verify the properties of MS as a *Tc*GS inhibitor: (i) E extracts were carried as described previously and incubated with different concentrations of MS at 28°C for 30 min. After this time, GS activity was measured. (ii) *Tc*GSr was purified as described previously, and the glutamine synthetase activity reaction was started with different concentrations of MS.

Moreover, we investigated the inhibition constant (K_i_) of glutamate. This constant is related to enzyme-inhibitor affinity. The K_i_ determination consists of the reestablishment of kinetic parameters under different concentrations of the inhibitor. Ultimately, K_i_ is the x-axis intersection of the linear function derived from the ratio of apparent V_max_ / apparent K_M_ [85].

### Growth inhibition assays

E of *T*. *cruzi* in the exponential growth phase (5.0 to 6.0 x 10^7^ cells/ml) cultured in fresh-LIT medium were treated with different concentrations of MS (range of 50 μM to 1,000 μM) or not (negative control). Rotenone (60 μM) and antimycin (0.5 μM) were used as positive controls. Cells (2.5 x 10^6^ cells/ml) were kept in 96-well culture plates at 28°C. Cell proliferation was estimated by reading the optical density (OD) at 620 nm for eight days as previously described [46]. On the fifth day of proliferation (exponential growth phase), the IC_50_ was calculated by fitting the data to a typical dose-response sigmoidal curve using the programs OriginPro8 and GraphPad Prism 5.0.

### Effect of MS in CDT bursting

CHO-K_1_ cells (5.0 x 10^4^ per well) were infected with CDT forms (2.5 x 10^6^ per well—50 parasites per cell) for three h in a 24 well-plate. CHO-K_1_ were cultivated in RPMI medium supplemented with SFB 10%, incubated at 37°C and treated with different concentrations of MS (range of 5 μM to 500 μM). The plate was incubated at 33°C. CDT were collected in the extracellular medium on the fifth day and counted in a Neubauer chamber.

### Effect of MS in parasitophorous vacuole evasion

CHO-K_1_ cells (1.0 x 10^4^ per dish—Corning BioCoat Culture Dishes, New York, NY, United States), cultivated in RPMI medium supplemented with SFB 10% were incubated with CytoPainter LysoNIR Indicator Reagent (Abcam, Cambridge, United Kingdom—1:1000 –v/v) for 1 h, LysoNIR is a lysotropic dye that selectively accumulates in lysosomes via the lysosome pH gradient. After this time, the cultures were washed 2 times with PBS and infected with CDT forms (5 x 10^5^ per well– 50 parasites per cell) for three h. The cultures were washed another 2 times with PBS and incubated with MS (concentrations for IC_50_ and IC_80_ in the intracellular cycle– 20) and Hoechst 33342 (1:1000 –Thermo Fisher Scientific). After different incubation times (T0; 1 h, 6 h and 24 h), images were acquired with a digital DFC 365 FX camera coupled to a DMI6000B/AF6000 microscope (Leica), and Software AF6000 was used to estimate the percentage of parasites within parasitophorous vacuoles.

## Supporting information

S1 Fig GS activity was measured in E cell-free extracts. The system of coupled reactions described in Materials and Methods was used to obtain the time-course curves at different concentrations for each of the three substrates: Glutamate (A), ATP (B) and NH_4_OH (C) (while keeping the others at saturating concentrations). Initial velocities (V_0_) at each concentration were used to construct the V_0_ vs [S] curves, allowing calculation of the kinetic parameters V_max_, K_M_ and K_cat_ for the enzyme. Data were adjusted to a Michaelis-Menten equation as described in Materials and Methods. The parameters are described in Table 1.
(TIF)

S2 Fig Specificity of Anti-GS antibody. (A) The recombinant protein was analyzed by SDS-PAGE using 10% (v/v) polyacrylamide gels under reducing conditions and visualized by Coomassie Blue staining. EC: Elutions concentrated by Amicon Ultra-4 50K (Millipore, Burlington, Massachusetts, United States). (B) Representative Western blot performed with EC against different dilutions of Anti-GS. (C) Representative Western blot performed with epimastigote extract (10 μg per well) against different dilutions of Anti-GS antibody.
(TIF)

S3 Fig Representative images of the effect of MS on evasion of the parasitophorous vacuole of *T*. *cruzi*. CHO-K_1_ cells were labeled with LysoNIR and then incubated with CDT to initiate infection. After 3 h, the cultures were washed and submitted to the treatment (or not). Nuclear DNA (N) was stained with Hoechst 33342. The cultures were observed and photographically registered. (A) Control untreated at 1 hour post-infection; (B) Control untreated at 24 hours post-infection; (C) Treatment with 20 μM MS (corresponding to EC_50_) at 1 hour post-infection; (D) Treatment with MS in EC_50_ concentration at 24 hours post-infection; (E) Treatment with 10 mM NH_4_Cl (as a positive control) at 1 hour post-infection; (F) Treatment with 10 mM NH_4_Cl at 24 hour post-infection. The black arrows are indicating the cells magnified in the right.
(PDF)
