# Supplementary figures and images for: The glutamine synthetase of Trypanosoma cruzi is required for its resistance to ammonium accumulation and evasion of the parasitophorous vacuole during host-cell infection
Source: PLoS Negl Trop Dis. 2018 Jan 10;12(1):e0006170. doi: 10.1371/journal.pntd.0006170 (PMC5779702; doi:10.1371/journal.pntd.0006170)

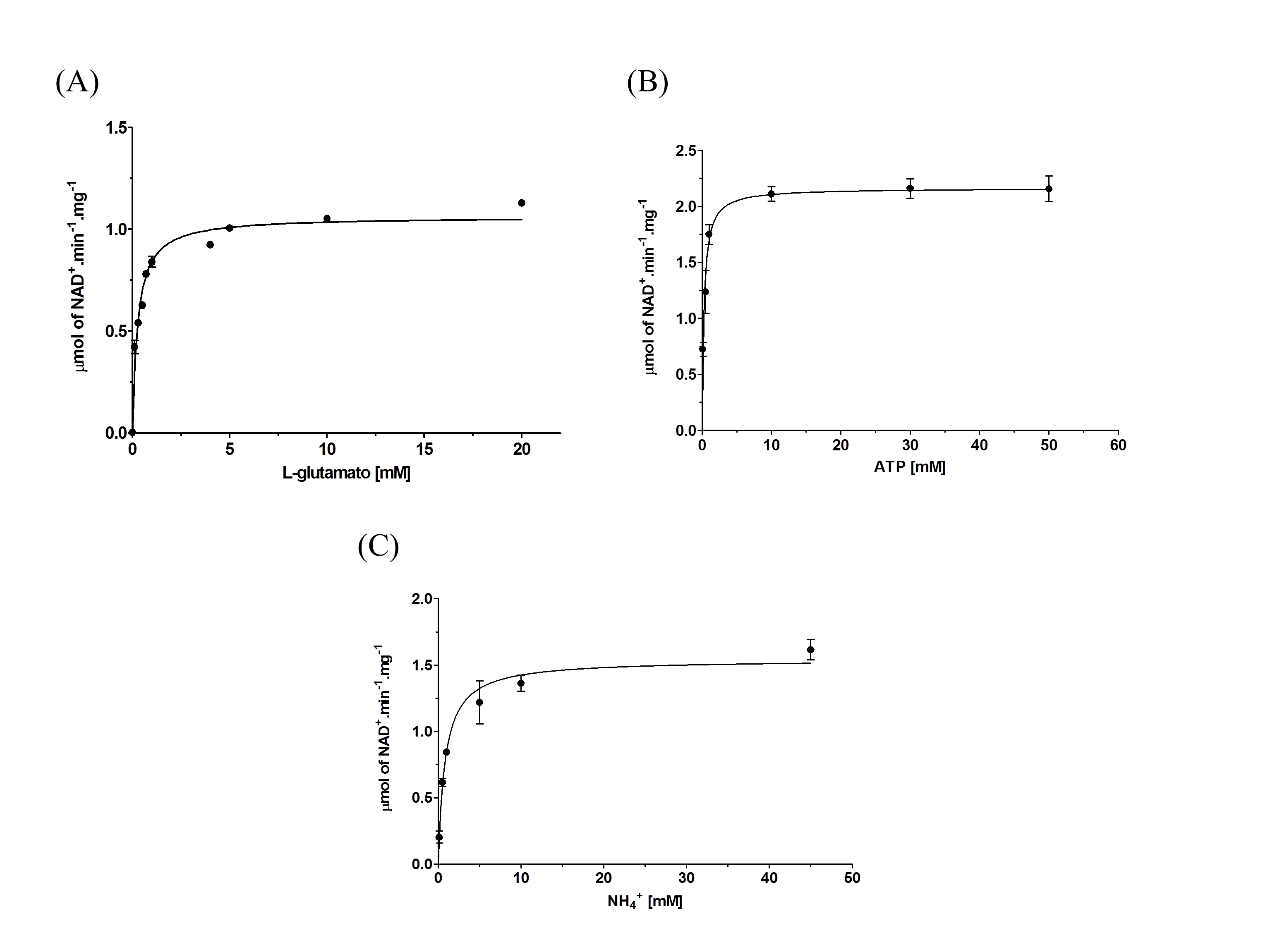

Supplement: S1 Fig — The system of coupled reactions described in Materials and Methods was used to obtain the time-course curves at different concentrations for each of the three substrates: Glutamate (A), ATP (B) and NH4OH (C) (while keeping the others at saturating concentrations). Initial velocities (V0) at each concentration were used to construct the V0 vs [S] curves, allowing calculation of the kinetic parameters Vmax, KM and Kcat for the enzyme. Data were adjusted to a Michaelis-Menten equation as described in Materials and Methods. The parameters are described in Table 1. (TIF) [file pntd.0006170.s001.tif]

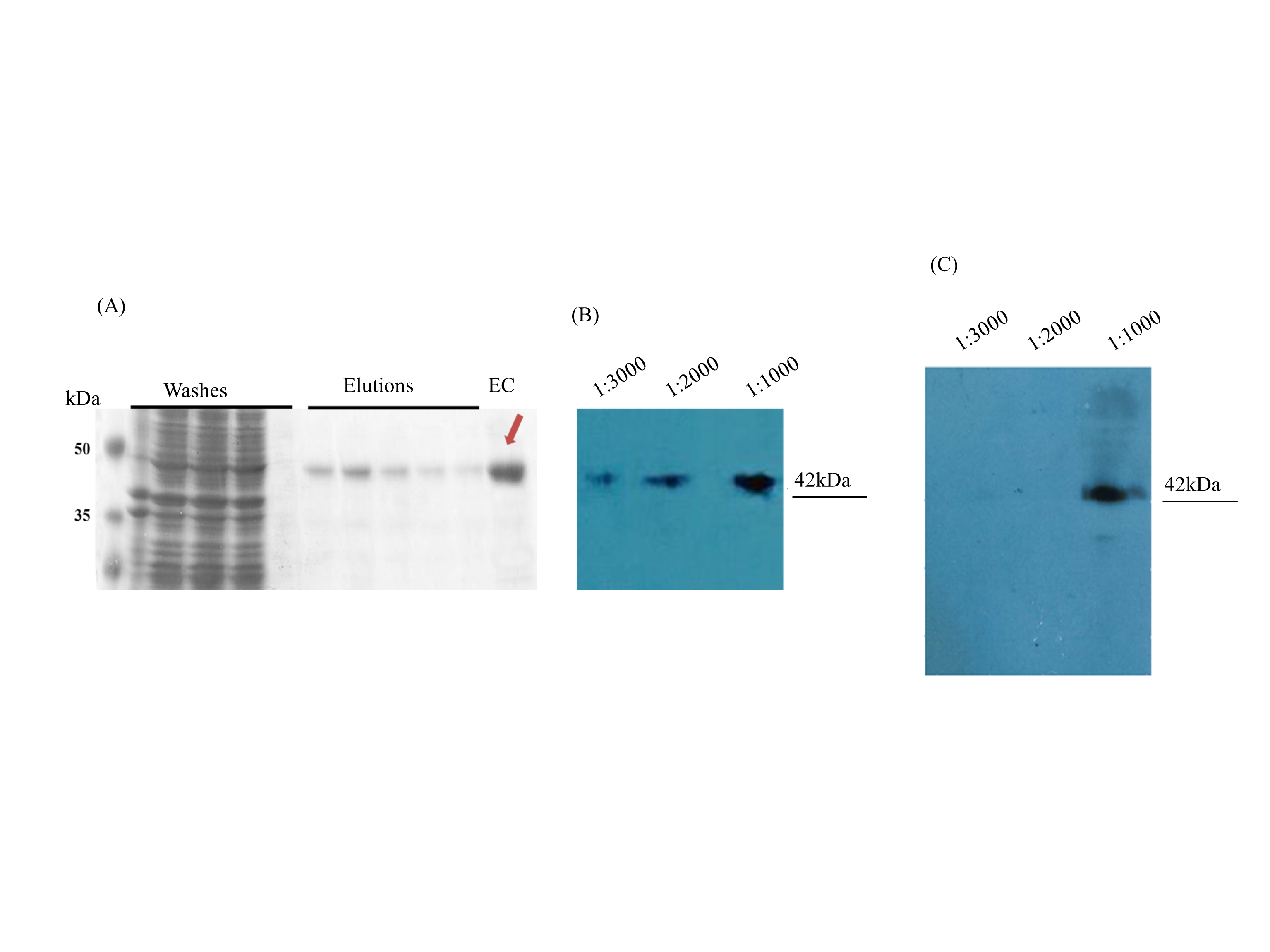

Supplement: S2 Fig — (A) The recombinant protein was analyzed by SDS-PAGE using 10% (v/v) polyacrylamide gels under reducing conditions and visualized by Coomassie Blue staining. EC: Elutions concentrated by Amicon Ultra-4 50K (Millipore, Burlington, Massachusetts, United States). (B) Representative Western blot performed with EC against different dilutions of Anti-GS. (C) Representative Western blot performed with epimastigote extract (10 μg per well) against different dilutions of Anti-GS antibody. (TIF) [file pntd.0006170.s002.tif]
