# Supplementary material for: The glutamine synthetase of Trypanosoma cruzi is required for its resistance to ammonium accumulation and evasion of the parasitophorous vacuole during host-cell infection
Source: PLoS Negl Trop Dis. 2018 Jan 10;12(1):e0006170. doi: 10.1371/journal.pntd.0006170 (PMC5779702; doi:10.1371/journal.pntd.0006170)

A

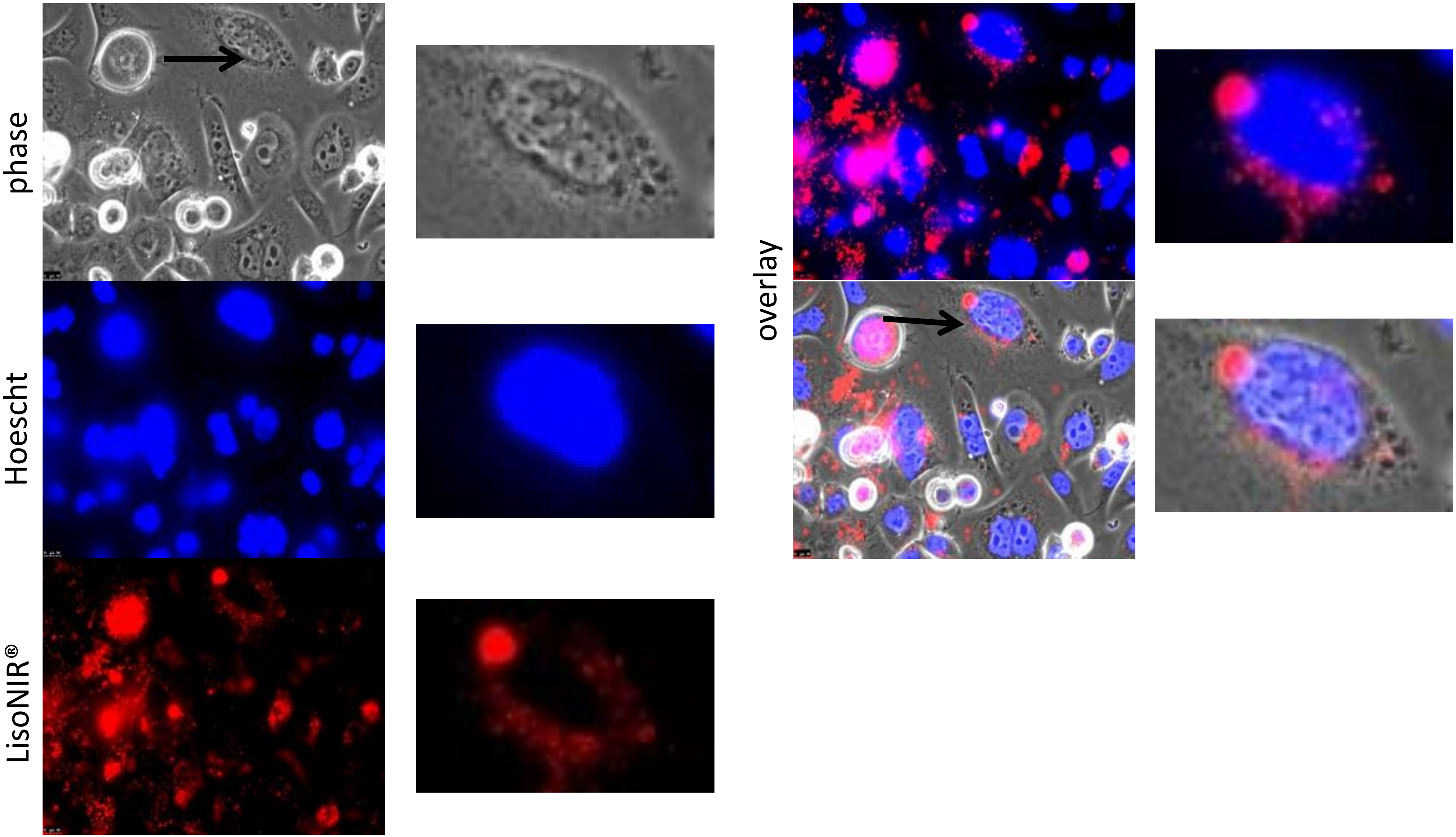

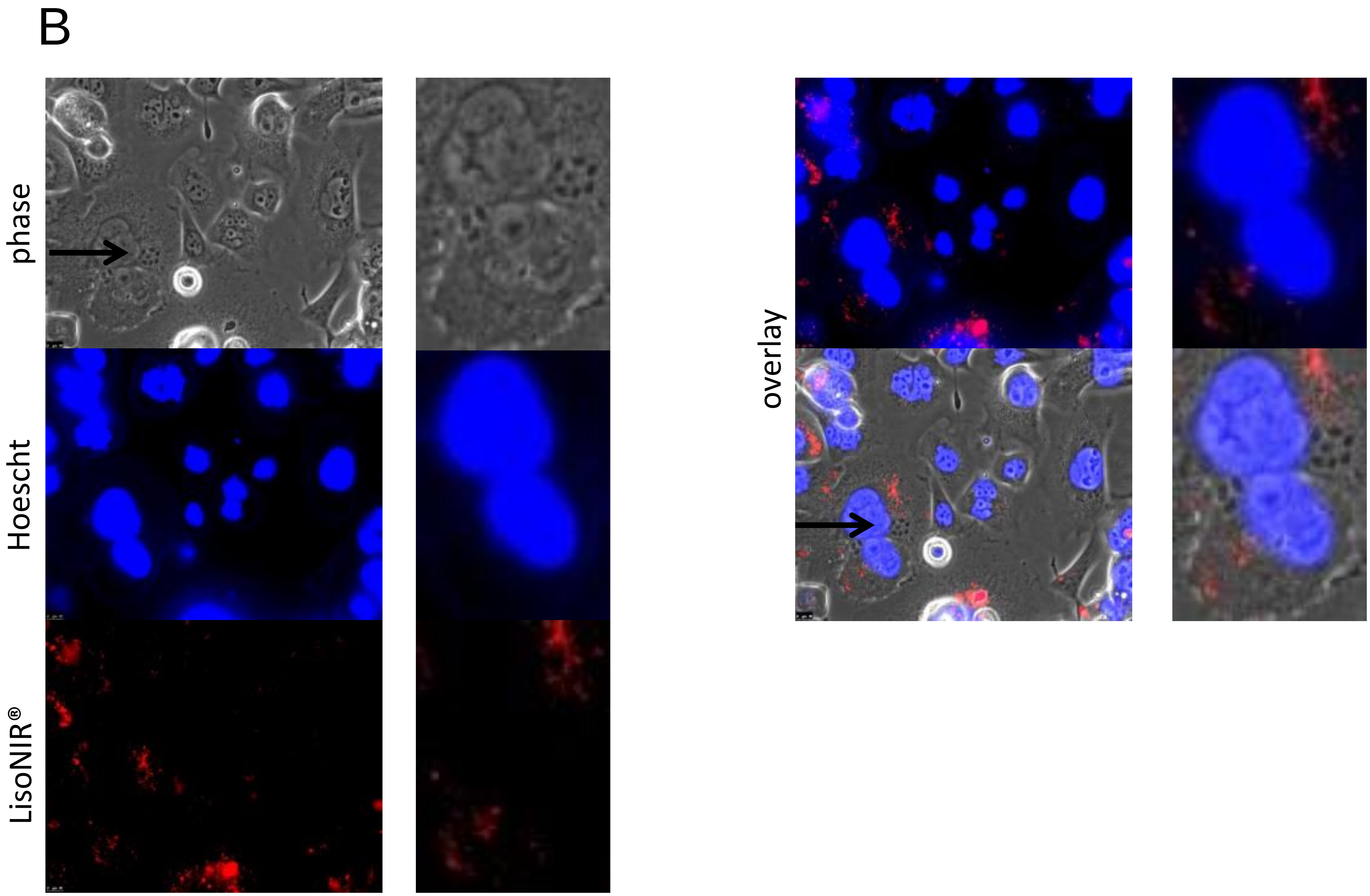
B
phase
Hoescht
LisoNIR®
overlay

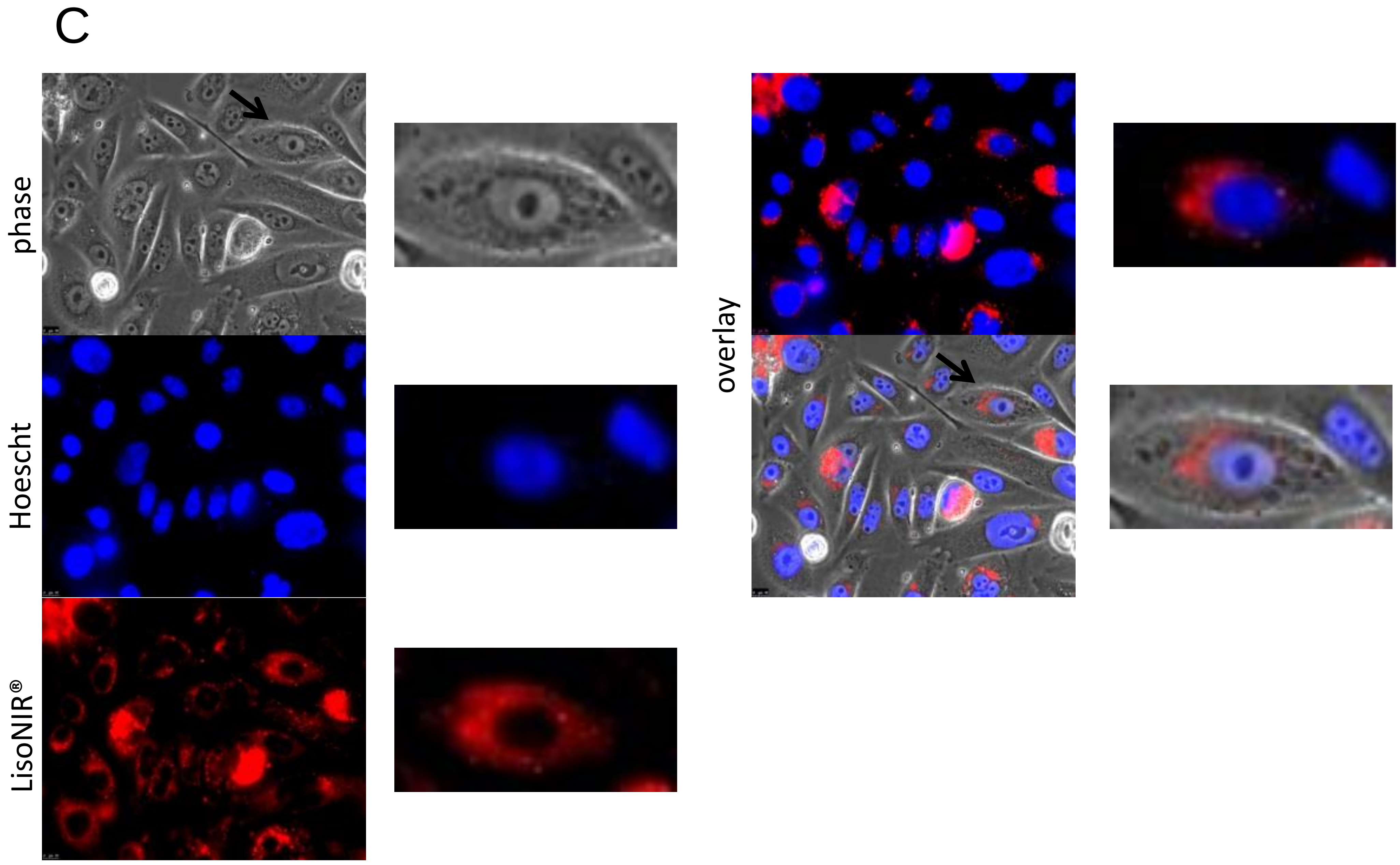
C
phase
Hoescht
LisoNIR®
overlay

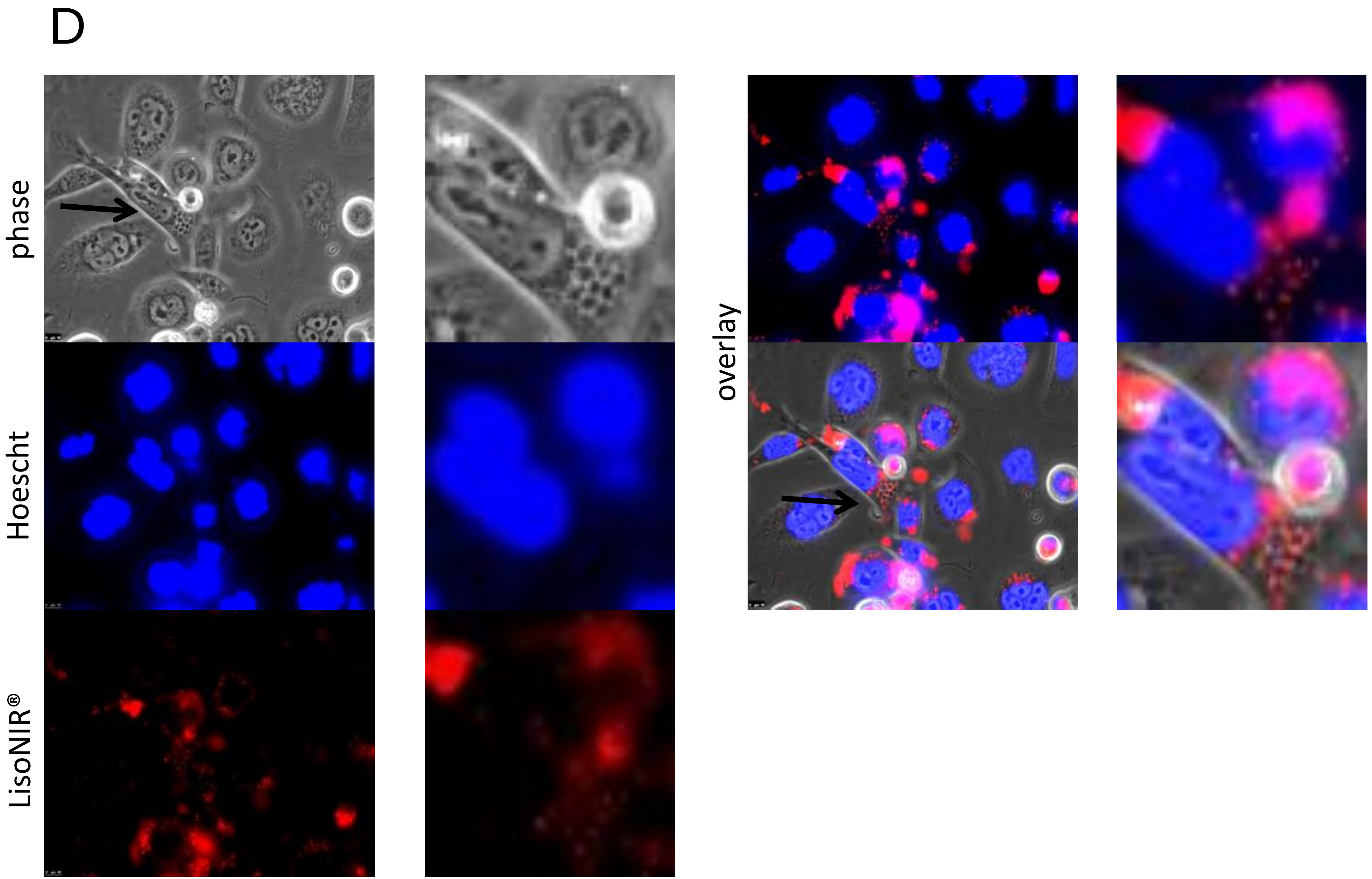
D
phase
Hoescht
LisoNIR®
overlay

E

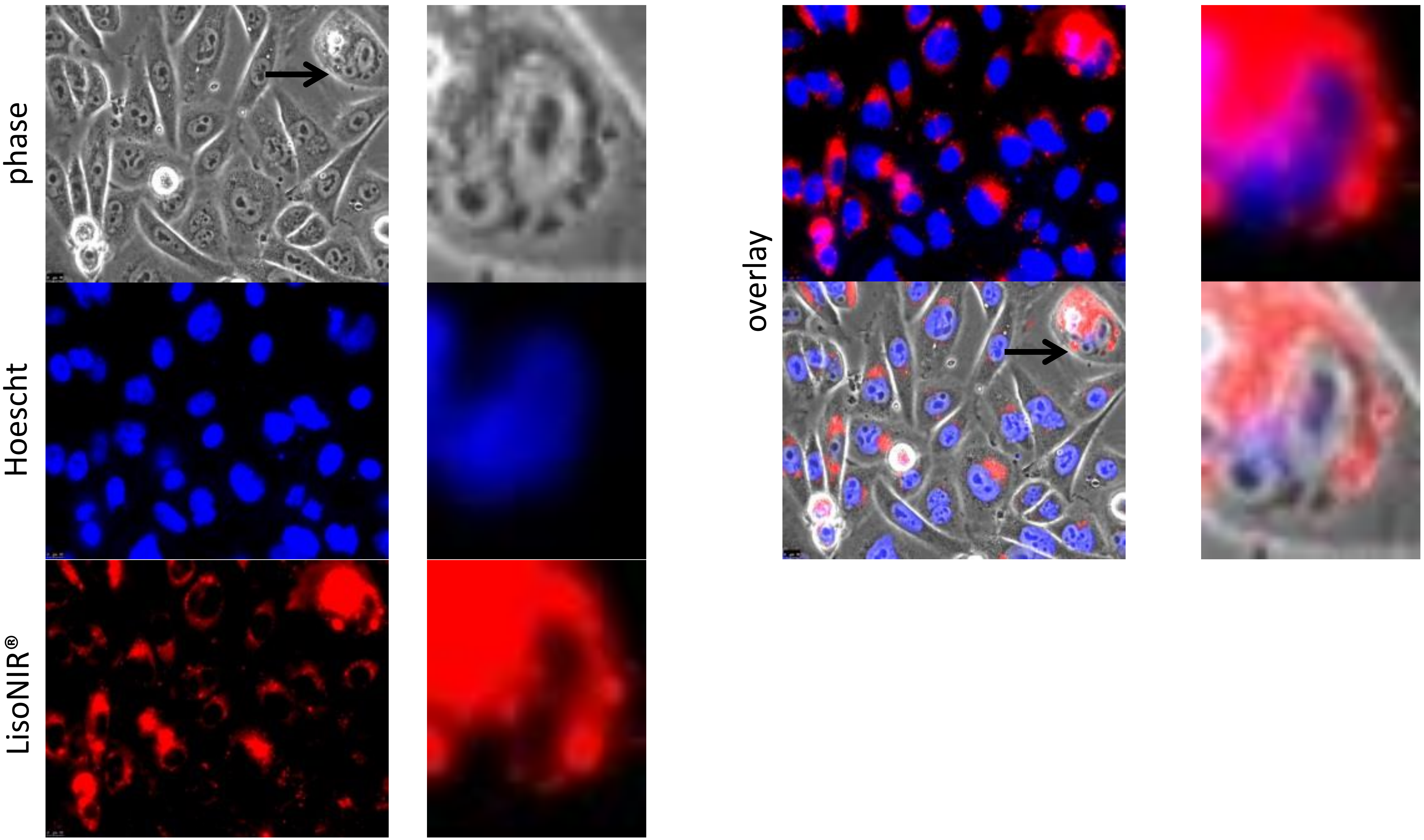

F

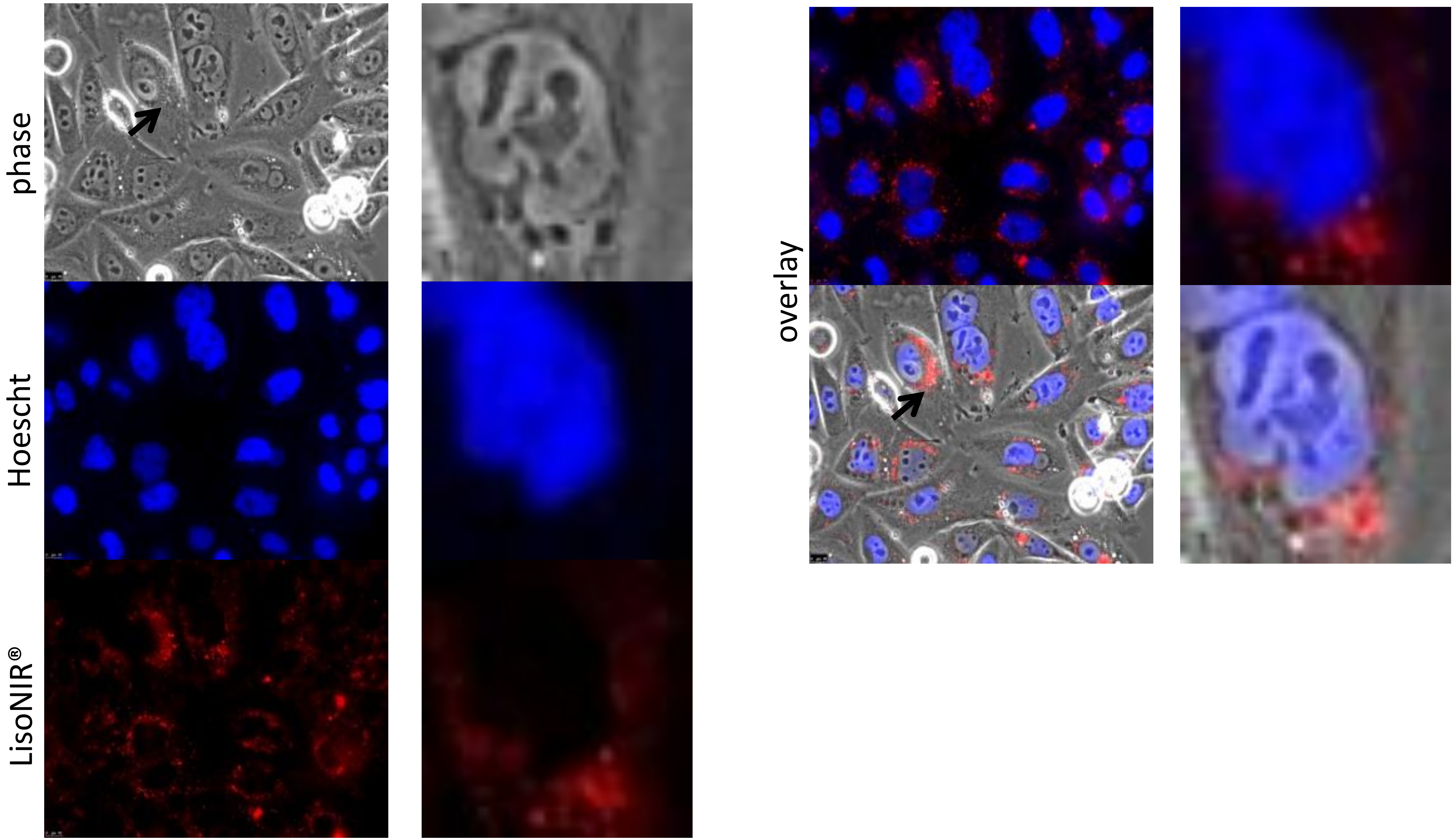

Supplement: S3 Fig — CHO-K1 cells were labeled with LysoNIR and then incubated with CDT to initiate infection. After 3 h, the cultures were washed and submitted to the treatment (or not). Nuclear DNA (N) was stained with Hoechst 33342. The cultures were observed and photographically registered. (A) Control untreated at 1 hour post-infection; (B) Control untreated at 24 hours post-infection; (C) Treatment with 20 μM MS (corresponding to EC50) at 1 hour post-infection; (D) Treatment with MS in EC50 concentration at 24 hours post-infection; (E) Treatment with 10 mM NH4Cl (as a positive control) at 1 hour post-infection; (F) Treatment with 10 mM NH4Cl at 24 hour post-infection. The black arrows are indicating the cells magnified in the right. (PDF) [file pntd.0006170.s003.pdf]
